# Nuclear Phosphoinositides as Key Determinants of Nuclear Functions

**DOI:** 10.3390/biom13071049

**Published:** 2023-06-28

**Authors:** Magdalena C. Vidalle, Bhavwanti Sheth, Antonietta Fazio, Maria Vittoria Marvi, Stefano Leto, Foteini-Dionysia Koufi, Irene Neri, Irene Casalin, Giulia Ramazzotti, Matilde Y. Follo, Stefano Ratti, Lucia Manzoli, Sonakshi Gehlot, Nullin Divecha, Roberta Fiume

**Affiliations:** 1 Inositide Laboratory, School of Biological Sciences, Faculty of Environmental and Life Sciences, University of Southampton, Life Sciences Building 85, Highfield, Southampton SO17 1BJ, UK; 2 Department of Biomedical Sciences (DIBINEM), University of Bologna, Via Irnerio 48, 40126 Bologna, Italy

**Keywords:** phosphoinositides, PtdIns(4,5)*P*_2_, signalling messengers, lipid, kinases, nucleus, epigenetic signalling, nuclear speckles, transcriptional output, mRNA machinery

## Abstract

Polyphosphoinositides (PPIns) are signalling messengers representing less than five per cent of the total phospholipid concentration within the cell. Despite their low concentration, these lipids are critical regulators of various cellular processes, including cell cycle, differentiation, gene transcription, apoptosis and motility. PPIns are generated by the phosphorylation of the inositol head group of phosphatidylinositol (PtdIns). Different pools of PPIns are found at distinct subcellular compartments, which are regulated by an array of kinases, phosphatases and phospholipases. Six of the seven PPIns species have been found in the nucleus, including the nuclear envelope, the nucleoplasm and the nucleolus. The identification and characterisation of PPIns interactor and effector proteins in the nucleus have led to increasing interest in the role of PPIns in nuclear signalling. However, the regulation and functions of PPIns in the nucleus are complex and are still being elucidated. This review summarises our current understanding of the localisation, biogenesis and physiological functions of the different PPIns species in the nucleus.

## 1. Introduction

**Figure 1:**
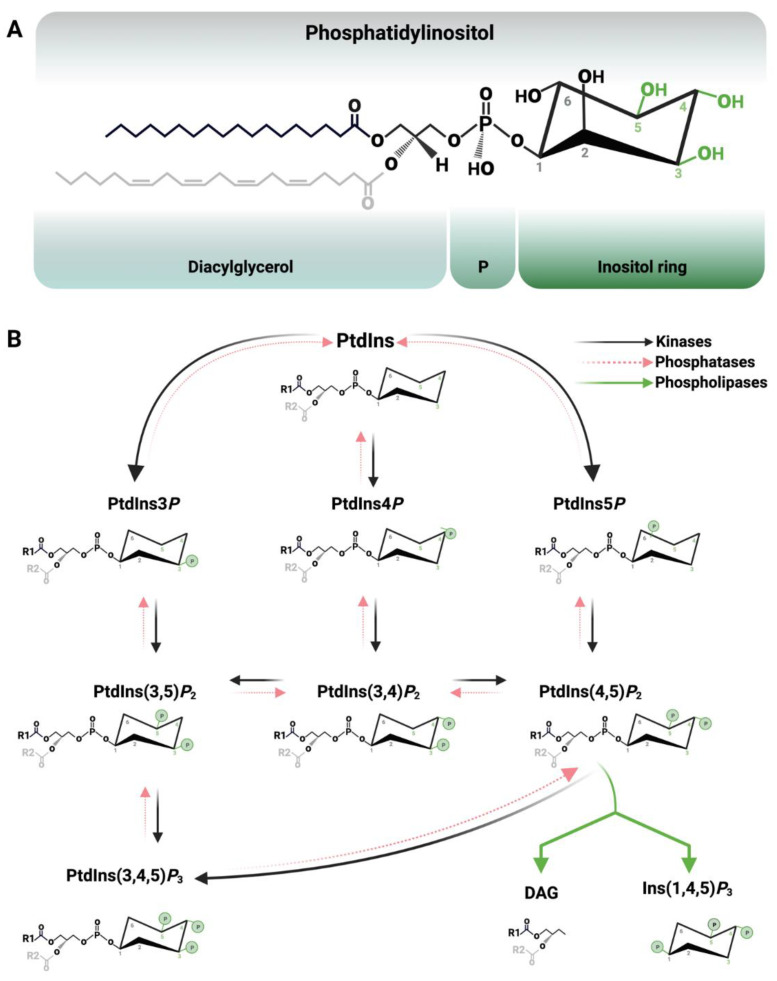
General structure and biogenesis of polyphosphoinositides (PPIns). (**A**). Phosphatidylinositol (PtdIns) structure as a C38:4 species, predominantly composed of a stearoyl acyl chain (C18:0) at the *sn*-1 (R1 in black) and an arachidonoyl (C20:4) acyl chain position at the *sn*-2 position (R2 in grey) [15,16,17]. The hydroxyl groups of the *myo*-inositol ring can be reversibly phosphorylated at positions C3, C4 and C5 (shown in green), leading to the formation of the different PPIns. (**B**). PPIns biogenesis, which is regulated through an array of kinases (black arrows), phosphatases (red arrows) and phospholipases (green arrows). The schematic is just a brief representation of some of the pathways that produce the different PPIns species. Abbreviations: P: phosphate, DAG: diacylglycerol.

Polyphosphoinositides (PPIns) constitute a group of phospholipids that act as signalling messengers and participate in a wide number of cellular processes. PPIns constitute only about 5% of the total cell phospholipid pool; however, these glycerol-based phospholipids are crucial regulators in orchestrating key biological signalling pathways, including cell division, vesicle transport, differentiation, autophagy, ion channel function and gene transcription [1,2]. The primary structure of PPIns is phosphatidylinositol (PtdIns), a *myo*-inositol hydrophilic head group linked to the *sn*-3 position of the glycerol group of a hydrophobic diacylglycerol tail via a phosphodiester bond [3,4,5] (Figure 1A). The resulting amphipathic properties of PPIns allow its positioning with the hydrophilic headgroup orientated towards the cytoplasm while the hydrophobic diacylglycerol tails are embedded in a lipid bilayer. The hydroxyl groups of the *myo*-inositol headgroup ring can be reversibly phosphorylated at 3, 4 and 5 position, leading to the formation of poly-acidic phospholipids [2,6]. Mono-, bis- and tris-phosphorylation by specific lipid kinases generate the seven PPIns species known to date: PtdIns3*P*, PtdIns4*P*, PtdIns5*P*, PtdIns(3,4)*P*_2_, PtdIns(3,5)*P*_2_, PtdIns(4,5)*P*_2_ and PtdIns(3,4,5)*P*_3_ (Figure 1B), which a panel of lipid phosphatases can dephosphorylate to form dynamically regulated subcellular pools of specific PPIns involved in the control of many cellular functions. For instance, a major mechanism by which PPIns control downstream signalling is their ability to recruit proteins to their surfaces by interacting with specific protein domains. For example, PPIns headgroups can be recognised by pleckstrin homology (PH) domains [7,8,9,10,11,12], with different PH domains showing specificity for different phosphoinositides. One of the most-well studied PH domains is that from phospholipase C-δ1 (PLC-δ1), which strongly and specifically binds the headgroup of PtdIns(4,5)*P*_2_, mediating the recruitment of PLC-δ1 to the plasma membrane and promoting the PLC-δ1 catalytic activity [13]. In contrast, the PH domain from GRP1 (general receptor for phosphoinositides, isoform 1) strongly and specifically interacts with PtdIns(3,4,5)*P*_3_ [14]. A table of known binding domains, together with their specificity for distinct PPIns, is shown in Table 1.

In contrast to the importance of the inositol head group, the importance of acyl chain specificity in modulating downstream signalling is still under debate. The fatty acyl chains of PPIns are generally found to be enriched in a stearoyl chain in *sn*-1 (18 carbons and no double bonds; C18:0) and an arachidonoyl chain in the *sn*-2 (20 carbons and 4 double bonds; C20:4). As these are quantitated by mass spectrometry of the intact lipid, they are often referred to as C38:4 [15,16,17] (Figure 1A). The strong enrichment of these fatty acyl species is uncommon, as most other phospholipids show a variety of alkyl/acyl compositions. How enrichment of these fatty acids is generated and maintained is still not clear but, given that the only mammalian PI-synthase shows no specificity for acyl chain composition, it is thought that fatty acyl remodelling through the Lands cycle is important [18,19]. In the Lands cycle, phospholipase-mediated removal of a fatty acyl group from a phospholipid generates a lyso-phospholipid, which can then be re-acylated, effectively remodelling the initial phospholipid. Moreover, while most tissues and cells show C38:4 enrichment in PPIns, others, such as testis and platelets, show very different acyl compositions, as do a number of cancer cell lines grown in culture. This has led to questions surrounding the importance of the acyl chain specificity of phosphoinositides in downstream signalling. The enzyme LPIAT (Lysophosphatidylinositol-acyl-transferase) is part of the Lands cycle, and can selectively reacylate LPI on the *sn*-2 position to incorporate arachidonyl. While knockouts of this enzyme in mice lead to neonatal lethality and aberrant brain development, it is not clear if this is due to reduced proportions of C38:4 PPIns (which was relatively minor), increased levels of LPI or a general decrease in the levels of PPIns [15,20]. This still leaves the question surrounding the importance of the fatty acyl chains in regulating downstream signalling. The distinct acyl composition could act as a molecular beacon to facilitate PtdIns resynthesis during receptor stimulation, or it may modulate the ability of effectors to interact with the head group and, indeed, there is data to support both hypotheses [21]. However, there is still a lack of biochemical and structural evidence to back up the evolutionary pressure that has driven the maintenance of this acyl chain specificity.

**Figure 2:**
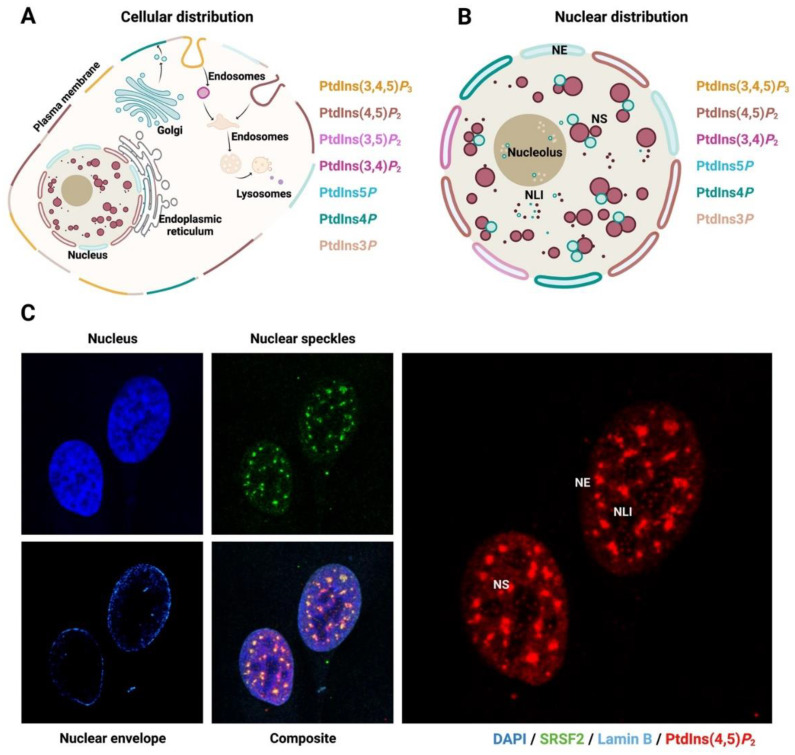
Nuclear localisation of polyphosphoinositides (PPIns). (**A**) Sub-cellular distribution of the different species of phosphoinositides. The colours show the different pools of phosphoinositides that can be found in different cell compartments, which are regulated through an array of kinases, phosphatases and phospholipases. (**B**) Nuclear structure and PPIn localisation. The schematic shows a brief representation of the PtdIns species found in the different nuclear sub-compartments. (**C**) Immunostaining of U20S cells showing nuclear localisation of PtdIns(4,5)*P*_2_. Nuclear speckles are stained with speckle protein SRSF2 (in green), the nuclear envelope is stained with Lamin B (in light blue) and PtdIns(4,5)*P*_2_ is stained with monoclonal antibody 2C11 [61]. PtdIns(4,5)*P*_2_ is found primarily at the nuclear speckles, nuclear envelope and nuclear lipid islets. Abbreviations: NE: nuclear envelope, NS: nuclear speckles, NLI: nuclear lipid islets.

The identification of protein domains capable of interacting with specific PPIns enabled the development of genetically encoded fluorescent probes to interrogate their subcellular localisation. The PLCδ1-PH domain can be used to specifically localise PtdIns(4,5)*P*_2_ [13], the PH domain of GRP1 labels PtdIns(3,4,5)*P*_3_ [14], while the multimerised FYVE domain from EEA1 (early endosome antigen-1) can localise PtdIns3*P* [22]. There are some caveats with respect to the use of these probes, as it is often unclear if they stably interact and label the specific PPIns in concert with their interaction with a different component, which may skew the localisation data. With this caveat in mind, the different PPIns species are found in various subcellular compartments, including the plasma membrane, the lysosomes, the Golgi, the endoplasmic reticulum and the nucleus (Figure 2A). The maintenance of specific PPIns pools within these compartments is likely a consequence of compartment-specific regulation of kinases, phosphatases and phospholipases (Figure 1B). Both internal and external stimuli impact the activity of these enzymes, leading to subcellular compartment-specific changes in PPIns, which eventually translates the stimuli into an effective operating output [2,6,23,24]. The amounts and distribution of the different PPIns pools within the cells are critical for normal cell development and function. Alterations to the levels of PPIns or their regulatory enzymes may directly influence the development of pathophysiological dysfunctions, including cancer, neuropathy and diabetes [25,26,27,28,29,30,31,32].

**Table 1:** List of phosphoinositide recognition protein domains.

Protein Domain	Phosphoinositide(s) Bound	References
Pleckstrin homology (PH) domain	PtdIns3*P*, PtdIns4*P*, PtdIns(4,5)*P*_2_, PtdIns(3,4)*P*_2_ PtdIns(3,4,5)*P*_3_	[10,11,12,13,14,33,34,35,36]
Phox homology (PX) domain	PtdIns3*P*, PtdIns(3,4)*P*_2,_ PtdIns(4,5)*P*_2_	[37,38,39]
Plant homeodomain (PHD)	PtdIns3*P*, PtdIns5*P*	[40,41]
FYVE domain	PtdIns3*P*	[42]
ENTH domain	PtdIns(4,5)*P*_2_	[43]
ANTH domain	PtdIns(4,5)*P*_2_	[44]
Polybasic domains	PtdIns(4,5)*P*_2_	[45,46,47]
Tubby	PtdIns(4,5)*P*_2_	[48,49]

## 2. The Nucleus and Nuclear PPIn Transport 

PPIns are normally maintained within membrane-bound structures due to their hydrophobic properties. However, PPIns are not only part of the nuclear envelope in the nucleus, they are also an important constituent of membrane-less nuclear bodies. To understand better, it is convenient to summarise the primary composition of the nucleus (Figure 2B,C). In eukaryotic cells, the nucleoplasm is separated from the cytoplasm by a double membrane bilayer known as the nuclear envelope (NE). Nuclear pore complexes (NPC) are distinctively structured focal apertures able to pierce the NE at spaced intervals and serve as transport channels between the cytoplasm and the nucleoplasm [50]. Proteins larger than about 40 KDa cannot directly diffuse through these pores and traffic to and from the nucleus through the presence of one or several characterised protein motifs known as nuclear localisation signals (NLSs) and a nuclear export signals (NESs) [51,52]. The subcellular localisation of proteins is tightly controlled by these conserved domains. The outer side of the NE is a continuation of the endoplasmic reticulum (ER) rich in ribosomes, while the inner side of the NE contains the so-called inner nuclear membrane proteins (INM proteins), including lamina-associated polypeptide 1 (LAP-1), lamin B receptor (LBR) and emerin, which interact with proteins of the nuclear lamina and with chromatin [53,54,55].

The composition of the nucleoplasm primarily comprises chromatin, RNA and nuclear proteins. Chromatin is the highly ordered organisation of the genome, which primarily consists of DNA (around 150 bp long) wrapped around a histone octamer made of two subunits from each histone, namely H2A, H2B, H3 and H4, to form the nucleosome. Histone H1 binds between the nucleosomes and acts to control transcription, protects the nucleosome from degradation and further condenses chromatin into a packed state. Changes in chromatin packing from a “closed” to an “open” higher-order chromatin conformation state impact on how DNA is utilised in processes such as DNA replication, RNA transcription and DNA repair. During interphase, each of the high-order chromatin portions, which comprise the different chromosomes, occupies discrete spatial regions within the nucleus known as the chromosome territories [56]. There are two distinct mechanisms that lead to chromatin conformational changes. The first mechanism is mediated by post-translational modifications of the core histones, including their methylation, acetylation, phosphorylation and ubiquitination, executed by a variety of enzymes (writers) that either act in directly modifying chromatin packing, as in the case of acetylation, or act in recruiting distinct epigenetic modulators (readers) that recognise and bind these modifications. Readers can recruit other proteins that translate these histone modifications into functional outputs. The second mechanism that leads to chromatin conformational changes is typically associated with the action of ATP-dependent chromatin remodelling complexes [57]. These complexes utilize the energy derived from ATP hydrolysis to remodel nucleosomes, alter chromatin structure and regulate access to DNA for various cellular processes, such as transcription, replication and repair. By using ATP hydrolysis, these complexes can alter nucleosome positioning, slide or evict nucleosomes and create DNA accessibility for other proteins. The ATP-dependent chromatin remodelling complexes include SWI/SNF (also known as BAF/PBAF), ISWI, CHD and INO80 families [57,58]. Besides the chromosome territories described, the nucleoplasm is also formed by interchromatin domains (ICD) rich in messenger RNA splicing factors, known as nuclear speckles [59,60]. Nuclear speckles are also membrane-less structures where pre-messenger RNA (pre-mRNA) machinery, including spliceosomes, small nuclear ribonucleoprotein particles (snRNPs) and other non-snRNP protein splicing factors, as well as factors required for DNA replication, transcription and repair are found [59]. Among the characteristic nuclear speckle factors are the arginine/serine-rich domain (RS domain)-containing proteins, including SRSF1 and SRSF2, which are key components for RNA splicing. The RS domain is sufficient for targeting these proteins to the nuclear speckle. Nuclear speckles are one of the main nuclear compartments where several species of PPIns have been observed [61,62].

There is clear evidence corroborating that, apart from nucleic acids and nuclear proteins, PPIns and their metabolising enzymes are also abundantly present in the nucleus [6,61,63,64,65,66,67,68]. How these different PPIns species are localised and controlled in the nucleus is still unclear. Are they formed in the cytoplasm and then translocated to the nucleus, or are they directly synthesised in the nucleus? Thus far, no PI-synthase has been found in the nucleus, suggesting that, at the least, PtdIns must be transported into the nucleus. PITPs are ubiquitous proteins found in most eukaryotic cells that can shuttle PtdIns within subcellular compartments. They contain a hydrophobic core that binds and shields the fatty acyl chains of PtdIns. The lipid can then be transferred to PtdIn-poor membranes where the lipid is swopped for a phosphatidylcholine (PC) molecule. Interestingly, two different variants of the PtdIn transfer proteins (PITPs) have been found in the nucleus [69,70,71]. PITPα shuttles between the cytoplasm and nucleus, while PITPβ is mainly localised to the perinuclear space, the region between the outer and the inner nuclear membrane [71]. However, knockout of PITPα did not significantly affect the levels of PtdIns present in nuclear fractions, as measured by mass spectrometry [72]. However, a very recent unpublished study [73] shows that loss of either PITPα or β reduces the levels of PtdIns(4,5)*P*_2_ at nuclear speckles measured by immune fluorescence, although whole cell PtdIns(4,5)*P*_2_ levels were not measured. These latter studies suggest a role for PITP in regulating nuclear PtdIns(4,5)*P*_2_, although this might occur at the level of regulating cytoplasmic PtdIns, with the defect being transport of PtdIns(4,5)*P*_2_ into the nucleus.

It could also be considered that nuclear PPIns are synthesised in the cytoplasm and translocated to the nucleus during cell division before the NE is formed. For instance, the pool of nuclear speckle PtdIns(4,5)*P*_2_ relocates to the cytoplasm during mitosis and aggregates in mitotic interchromatin granules (MIGs) that remain in the cytoplasm of the daughter cells after the NE is formed, and there is a progressive translocation into the nucleus of MIGs components during telophase [61,74]. Furthermore, there is a pool of nuclear PtdIns(4,5)*P*_2_ present in nucleolar organising regions (NORs) during mitosis that appears to remain in the nucleus after cell division is concluded [75]. Additionally, PPIn-metabolising enzymes, including kinases, phosphatases and phospholipases, have been found in the nucleus, which suggests that there is a PPIns synthesis pathway in the nucleus independent from the one in the cytoplasm.

## 3. Biogenesis of Nuclear Phosphoinositides and Their Metabolising Enzymes

In the early years of the PPIn research field, it was assumed that PPIns’ metabolism was mainly confined to the cytoplasm, with the large majority occurring at the plasma membrane. The first study to identify the presence of PPIn activity in the nucleus was carried out in isolated nuclear envelopes from a rat liver [76]. This study showed that isolated nuclear envelopes incubated with [γ-^32^P]ATP produced ^32^P-labelled PtdIns(4,5)*P*_2_ and phosphatidic acid (PA). This was the first insight into the existence of nuclear PtdIn metabolism. Following these findings, a biochemical study using highly purified nuclei from Friend erythroleukemia cells showed that the presence of PPIns was not limited to the nuclear envelope [67]. Nuclei isolated in the presence of Triton to remove the nuclear envelope resulted in the production of ^32^P-radiolabelled PtdIns(4,5)*P*_2_, PtdIns4*P* and PA after incubation with [γ-^32^P]ATP. These data show that PtdIns, PtdIns4*P* and DAG, along with kinases that phosphorylate them, are present within the nuclei. Interestingly the amount of radiolabelling of these lipids was dependent on the differentiation status and differed from that observed in isolated plasma membrane fractions. These data suggested that nuclear PPIns and DAG can be regulated independently from cytoplasmic PPIns. Subsequent studies demonstrated that IGF1 could regulate a nuclear PLC which hydrolysed nuclear PtdIns(4,5)*P*_2_ to generate nuclear DAG that caused the translocation of protein kinase C to the nucleus [64]. In contrast, treating cells with the regulatory tetra-decapeptide bombesin induced plasma membrane PtdIns(4,5)*P*_2_ hydrolysis and subsequence membrane translocation of PKC. These data showed that nuclear PPIns’ metabolism was distinctly regulated compared to its plasma membrane counterpart. Furthermore, immunofluorescence showed that PIP5Ks and its product PtdIns(4,5)*P*_2_ were present at nuclear speckles and colocalised with the splicing factor SRSF2 [68]. Moreover, fractionation studies suggested that nuclei contained two pools of PIP5Ks in the nucleus: a soluble pool, easily extracted using detergent, and a second pool that was detergent resistant, likely associated with the nuclear speckles [68]. Most interestingly, the presence of PIP5Ks and PtdIns(4,5)*P*_2_ was not associated with invaginations of the nuclear envelope or any membrane-containing organelle. These findings strongly indicate a novel pathway for the production of phosphoinositides within nuclei.

These were some of the early studies that set the foundation for the existence of a nuclear PPIns signalling network independent of the well-established site at the plasma membrane. However, many questions remain unaddressed. For instance, are nuclear PPIns synthesised in the nucleus and/or is there a trafficking event that brings PPIns from the cytoplasm into the nucleus? Assuming there is an independent PPIns metabolism pathway in the nucleus, which are the exact PPIns enzymes that regulate nuclear PPIns? Here, we describe the PPIns species that have been discovered in the nucleus so far and how they might be metabolised in the nucleus. Except for PtdIns(3,5)*P*_2_, all the PPIns species have been found in the nucleus [23,77].

### 3.1. PtdIns3P

The discovery of the zinc-finger-like domain, FYVE (Fab1p, YOTB, Vac1 and EEA1)-finger, as a PtdIns3*P* binding module was a key step in studying its sub-compartmental localisation and function [78,79]. Biochemical and morphological assays allowed the characterisation of a new probe that binds specifically and with high affinity to PtdIns3*P*, designed by multimerising a PtdIns3*P*-binding FYVE-finger domain from the receptor tyrosine kinase (RTK) substrate Hrs [78]. Overexpression of the double FYVE-finger domain and a double mutant FYVE^C215S^-finger domain, which did not bind PtdIns3*P*, were used in primary human fibroblasts and baby hamster kidney (BHK) fibroblasts cells [80]. High labelling of PtdIns3*P* within the dense fibrillar component of the nucleoli with the double FYVE-finger but not the mutant was observed in vitro and, for the first time, the nuclear localisation of PtdIns3*P* was unveiled [80]. The synthesis and turnover of PtdIns3*P* can be controlled following different routes. For instance, the inositol polyphosphate-4-phosphatase type I A (INPP4A) and inositol polyphosphate-4-phosphatase type I B (INPP4B) catalyse the hydrolysis of PtdIns(3,4)*P*_2_ at position 4 of the *myo*-inositol ring to generate PtdIns3*P* [81,82], while phosphorylation of PtdIns at position 3 of the *myo*-inositol ring catalysed mainly by the class II and III PtdIns 3-kinase enzymes (PI3KC2 and PI3KC3) [83,84]. PI3KC2 can generate PtdIns3*P* and PtdIns(3,4)*P*_2_ and are involved in active cell metabolism promoting cell growth and proliferation [85]. PI3KC2α is present in nuclear speckles [86], and PI3KC2β was shown to be activated in nuclei of HL60 cells after ATRA (all-trans-retinoic acid) mediated differentiation [87] or in response to EGF stimulation [88]. Moreover, PtdIns3*P* turnover can be controlled by the PtdIns 5-kinase FYVE-type zinc finger-containing (PIKfyve) family and the myotubularins (MTMs) family of phosphatases [89,90,91], although it remains unclear whether they regulate PtdIns3*P* in the nucleus.

### 3.2. PtdIns4P

In the nucleus, the presence of PtdIns4*P* has been characterised both biochemically [64,67,92] and by immunolocalization, where it is mainly found at the NE, in the nucleoli and in small foci in the nucleoplasm, probably nuclear speckles [62]. Moreover, numerous nuclear proteins have been identified which can interact with PtdIns4*P* [93]. There are three main pathways by which PtdIns4*P* is synthesised, including phosphorylation of PtdIns at position 4 of the *myo*-inositol ring by phosphatidylinositol 4-kinase (PI4K) or dephosphorylation of PtdIns(4,5)*P*_2_ or PtdIns(3,4)*P*_2_. Removal of PtdIns4*P* is mainly regulated by Sac1 at the endoplasmic reticulum (ER) membranes [94,95]. Exactly how PtdIns4*P* in the nucleus is regulated is still unclear.

### 3.3. PtdIns5P

Out of the seven PPIns species, PtdIns5*P* was the last one to be discovered, and represents less than 0.5% of the total amount of PPIns in mammalian cells [96,97]. Biochemical analyses aimed at understanding PtdIns(4,5)*P*_2_ synthesis discovered the existence of two classes of phosphatidylinositol-4-phosphate 5-kinase (PIP5K) enzymes, type I and type II, believed to phosphorylate PtdIns4*P* at position 5 of the *myo*-inositol ring to produce PtdIns(4,5)*P*_2_ [98,99,100]. When the substrate specificities of these enzymes were reanalysed in the late 1990s, it was found that type I enzymes phosphorylate PtdIns4*P* at position 5 of the *myo*-inositol ring, PIP5Ks, while type II enzymes phosphorylate PtdIns5*P* at position 4 of the *myo*-inositol ring, PIP4K [101]. There are three PIP4K isoforms in mammals: PIP4K2α, PIP4K2β and PIP4K2γ [102,103,104], which are thought to regulate the levels of specific pools of PtdIns5*P* and PtdIns(4,5)*P*_2_ [40,105,106,107,108,109,110,111,112,113]. The observation that PIP4K2β is present in the nucleus [106] led to the identification of PtdIns5*P* as a cell cycle [92] and stress induced nuclear lipid that controls epigenetic signalling [40,107,112,114,115].

PtdIns5*P* can be synthesised by PIKfyve kinase, which phosphorylates PtdIns at position 5 of the *myo*-inositol ring [116]. In addition, PIKfyve can also indirectly generate PtdIns5*P* via an alternative pathway by phosphorylating PtdIns3*P* at position 5 to generate PtdIns(3,5)*P*_2_, with subsequent dephosphorylation at position 3 by a 3-phosphatase [117,118]. PtdIns5*P* can also be produced by dephosphorylation of PtdIns(4,5)*P*_2_ by the activity of type I and type II PtdIns(4,5)P_2_-4-phosphatases in vitro [119] and in the nucleus, this route can regulate PtdIns5*P* levels [120].

### 3.4. PtdIns(3,4)P_2_

The absence of a probe that specifically binds PtdIns(3,4)*P*_2_ made the study of its localisation within the cell challenging. However, the spatially defined location of the different enzymes that catalyse the formation of PtdIns(3,4)*P*_2_ suggests the existence of different pools of this PPIns within the cell [84,121,122]. The development of a monoclonal antibody to PtdIns(3,4)*P*_2_ allowed the detection of this PPIns for the first time in the nuclear envelope [123]. There are two main substrates that yield PtdIns(3,4)*P*_2_: PtdIns4*P* and PtdIns(3,4,5)*P*_3_. Phosphorylation of PtdIns4*P* at position 3 of the *myo*-inositol ring is catalysed primarily by class II phosphatidylinositol-4-phosphate 3-kinase. However, the subcellular localisation of these kinases seems to define substrate specificity and their distinct roles. For instance, growth factor depletion induces PI3KC2 isoform β (PI3KC2β) to synthesise a specific subcellular pool of PtdIns(3,4)*P*_2_, which culminates with the repression of mammalian target of rapamycin complex 1 (mTORC1) function at lysosomes [124,125]. Dephosphorylation of PtdIns(3,4,5)*P*_3_ at position 5 of the *myo*-inositol ring is the alternative pathway to generate PtdIns(3,4)*P*_2_. Two main sets of phosphatases catalyse this reaction: the SH-containing inositol 5′-polyphosphatases (SHIP) family [126,127] and inositol polyphosphate-5-phosphatase J [128], with the latter having only shown in vitro activity with PtdIns(3,4,5)*P*_3_ as a substrate. Turnover of PtdIns(3,4)*P*_2_ is primarily regulated by inositol polyphosphate 4-phosphatase A (INPP4A) and B (INPP4B) to generate PtdIns3*P* by hydrolysing PtdIns(3,4)*P*_2_ at position 4 of the *myo*-inositol ring [125,129].

### 3.5. PtdIns(4,5)P_2_

PtdIns(4,5)*P*_2_ is the most abundant bis-phosphorylated PPIn in the cell, with the plasma membrane as its prime location. Extensive research has unveiled the presence of this PPIn species in the nucleus and its importance in nuclear signalling pathways. The presence of PtdIns(4,5)*P*_2_ in the nucleus has been shown biochemically [64,66,67] and through the use of commercially available antibodies [61,130]. There are currently three monoclonal antibodies against PtdIns(4,5)*P*_2_: clone AM212 [131], clone KT10 [132] and clone 2C11 [133]. Unfortunately, pleckstrin homology (PH) domains that bind with high specificity to PtdIns(4,5)*P*_2_, such as the PH domain of PLCδ1, cannot be used to dynamically measure nuclear PtdIns(4,5)*P*_2_ in live cells, although this has been successfully carried out for plasma membrane PtdIns(4,5)*P*_2_ [62].

PtdIns(4,5)*P*_2_ has been found at different locations within the nucleus, including nuclear speckles, nucleoli and the NE [130] (Figure 2B). Despite its amphipathic properties, part of the nuclear PtdIns(4,5)*P*_2_ pool is retained in membrane-less structures, such as nuclear speckles and in nucleosomes [61,134]. The localization of PtdIns(4,5)*P*_2_ in nuclear-membrane-less bodies may be the reason why the PH domain does not appear to stably associate with this nuclear pool of PtdIns(4,5)*P*_2_. Enzymes that regulate the biogenesis of PtdIns(4,5)*P*_2_ have been found in the nucleus and in nuclear speckles [64,65,68,135,136]. The synthesis of PtdIns(4,5)*P*_2_ is mainly catalysed by the phosphorylation of PtdIns4*P* at the fifth hydroxyl of the inositol ring by the family of type I PIP5K [101,137,138]. There are three PIP5K isoforms: PIP5K1α, PIP5K1β and PIP5K1γ [139,140] and three PIP4K isoforms: PIP4K1α, PIP4K1β and PIP4K1γ, all of which could regulate nuclear PtdIns(4,5)*P*_2_ [129]. In addition, dephosphorylation of PtdIns(3,4,5)*P*_3_ at position 3 of the inositol ring is an alternative pathway to produce PtdIns(4,5)*P*_2_, and is catalysed by an array of phosphatases, including PTEN and TPIP (TPIPα, -β and -γ) [141]. Two main families of enzymes regulate the hydrolysis of PtdIns(4,5)*P*_2_: the phospholipase C (PLC) family and the PtdIns(4,5)*P*_2_-5-phosphatase family, including INPP5E, INPP5J, INPP5B and SYNJ1 [142,143]. Although these enzymes have been found mainly in the cytoplasm, several studies have shown that PIP5Kα, PIP4K2β, PLCβ1 and PLCδ4 are present in the nucleus [68,92,138,144].

### 3.6. PtdIns(3,4,5)P_3_

The amount of PtdIns(3,4,5)*P*_3_ in mammalian cells is less than 0.05% of the total pool of PPIns. Several enzymes that regulate PtdIns(3,4,5)*P*_3_ levels were found in the nucleus, suggesting that PtdIns(3,4,5)*P*_3_ or a metabolite may have a nuclear role [145,146,147,148,149]. GRP1-PH is a specific PtdIns(3,4,5)*P*_3_ interactor [14] and was used to assess the subcellular distribution of PtdIns(3,4,5)*P*_3_ in cells using electron microscopy [147]. Upon PDGF stimulation, the levels of PtdIns(3,4,5)*P*_3_ in the plasma membrane increased as expected. Surprisingly, a similar increase in nuclear matrix associated PtdIns(3,4,5)*P*_3_ was also observed [147]. PtdIns(3,4,5)*P*_3_ is synthesised mainly by class I PtdIns3P kinases (PI3Ks) that catalyse the phosphorylation of PtdIns(4,5)*P*_2_ at position 3 of the inositol ring. There are four isoforms of class I PI3K in mammalian cells: class I PI3Kα, PI3Kβ, PI3Kδ and PI3Kγ; and their roles in the nucleus have been associated with major cellular processes, including cell cycle, differentiation and proliferation [150]. IPMK1 (inositol polyphosphate multikinase) can also contribute to PtdIns(3,4,5)*P*_3_ synthesis [151,152]. IPMK is found in the nucleus and possesses an evolutionarily conserved phosphoinositide 3-kinase activity. In the nucleus, IPMK phosphorylates PtdIns(4,5)*P*_2_ at position 3 of the inositol ring to produce PtdIns(3,4,5)*P*_3_ [151]. PtdIns(3,4,5)*P*_3_ turnover is primarily controlled by the PTEN and SHIP2 phosphatases which hydrolyse PtdIns(3,4,5)*P*_3_ at position 3 and position 4 of the inositol ring to generate PtdIns(4,5)*P*_2_ and PtdIns(3,5)*P*_2_, respectively [153,154,155]. Surprisingly, the expression of PTEN in the nucleus did not decrease PDGF-induced increases in PtdIns(3,4,5)*P*_3_, while its expression decreased plasma membrane PtdIns(3,4,5)*P*_3_, suggesting that nuclear PtdIns(3,4,5)*P*_3_ is not a good substrate for PTEN [147]. The main kinases and phosphatases that control the biogenesis of PtdIns(3,4,5)*P*_3_ have been associated with major human pathologies.

## 4. Physiological Functions of Nuclear Phosphoinositide

Changes in nuclear PPIns are observed in response to growth factors and stress signalling, cell cycle progression, DNA damage and cell differentiation. Identification of proteins that interact and transduce changes in nuclear PPIns into functional outputs, suggest they play important roles in the control of epigenetic signalling, DNA damage signalling, transcription factor regulation and RNA maturation and export, culminating in the regulation of gene transcriptional output and control cell fate decisions.

Modifications of the terminal histone tails of core nucleosomal histones include phosphorylation, methylation, acetylation and ubiquitination, and are among the epigenetic events that modulate gene transcription without altering the primary DNA sequence [156]. For instance, tri-methylation of the histone H3 lysine 4 (H3K4me3) or acetylation of lysine 4 (H3K4ac) at or near promoters of genes induce unpacking of the histone from the negatively charged DNA by reducing the positive charge of histones [157,158], and are associated with activation of gene transcription. Nuclear PPIns have been implicated in controlling many aspects of epigenetic signalling, as illustrated below.

### 4.1. Nuclear PPIns as Regulators of Histone Modifications

The transcriptional regulator Wilm’s tumour 1 protein (WT1) is involved in cell cycle progression and differentiation, and functions as both an activator and repressor of gene transcription [159]. The functional switch to a repressor occurs in part through interaction of WT1 with the membrane-bound brain acid soluble protein 1 (BASP1) [160,161]. The interaction requires the association of WT1 with the N-terminal myristoylated region of BASP1, which interacts with nuclear PtdIns(4,5)*P*_2_. The interaction between BASP1 and PtdIns(4,5)*P*_2_ recruits the histone deacetylase 1 protein (HDAC1) to promoters of WT1 target genes, leading to their transcriptional repression. The association between BASP1 and PtdIns(4,5)*P*_2_ is critical for BASP1 gene-specific corepressor-mediated activity with WT1 [162].

Conversely, ATX1 is a plant trithorax factor which catalyses tri-methylation of lysine 4 of histone H3 (H3K4me3) at selective gene promoters leading to active gene transcription. ATX1 contains a PHD finger which interacts with PtdIns5*P* and in response to drought stress, increased PtdIns5*P* promotes ATX1 translocation from the nucleus to the cytoplasm with a consequent decrease in H3K4me3 at specific gene promoters [115]. The ability of nuclear PPIns to directly regulate histone H3K4me3 may be a conserved action in Drosophila, as Skittles (a drosophila PIP5K), an enzyme that synthesises PtdIns(4,5)*P*_2_ interacts with Ash2, a core component of the H3K4-trimethylation complex in Drosophila [163].

Transcription can also be controlled by modulating chromatin structure through control of nucleosomal positioning regulated by chromatin remodelling complexes. One such complex is the BAF complex, which controls gene expression during T-cell development. The BAF complex interacts with nuclear PtdIns(4,5)*P*_2,_ which stabilises its interaction with chromatin, increasing the interaction of the BAF complex component, BRG1, with nuclear actin [164]. The functional repercussion of PtdIns(4,5)*P*_2_ binding the BAF-actin complex remains unclear; however, nuclear actin has roles in transcription [164,165]. Interestingly, a number of nuclear remodelling complexes have been shown to be regulated through their interaction with higher phosphorylated inositols, which are derived in part from the phosphorylation of Ins(1,4,5)*P*_3_ generated by PLC-mediated hydrolysis of PtdIns(4,5)*P*_2_ (for a review, see reference [105]).

Recent studies also suggest a novel mechanism by which nuclear PPIns might impact on transcriptional output. In response to changes in interactions between cells and the extracellular matrix, mechano-sensation leads to intracellular signalling changes that impact on nuclear morphology and transcriptional output. Changes in nuclear morphology have dramatic impacts on transcriptional output, as exemplified by human mutations in genes that form or control the nuclear lamina leading to early-onset ageing syndromes [166]. How exactly morphological changes impact on transcriptional output is not clear. This new study demonstrated that softening or decreasing matrix interactions induces the degradation of PIP4K2β, leading to increased nuclear PtdIns5*P* levels. This was associated with decreased YAP signalling and increased trimethylation at H3K9 (H3K9me3), which is associated with transcriptional repression. Further studies are required to understand the exact molecular details that link increased nuclear PtdIns5*P*, YAP signalling and H3K9me3 [112].

In a more direct manner, PtdIns(4,5)*P*_2_ can interact with Histone H1 and H3 tails that are positively charged, and this interaction can reverse H1-mediated inhibition of transcriptional activity [167]. Whether this plays a role in vivo is not clear.

### 4.2. Nuclear PPIns and Their Role in Defining How Histone Modifications Drive Downstream Signalling Outputs

**Figure 3:**
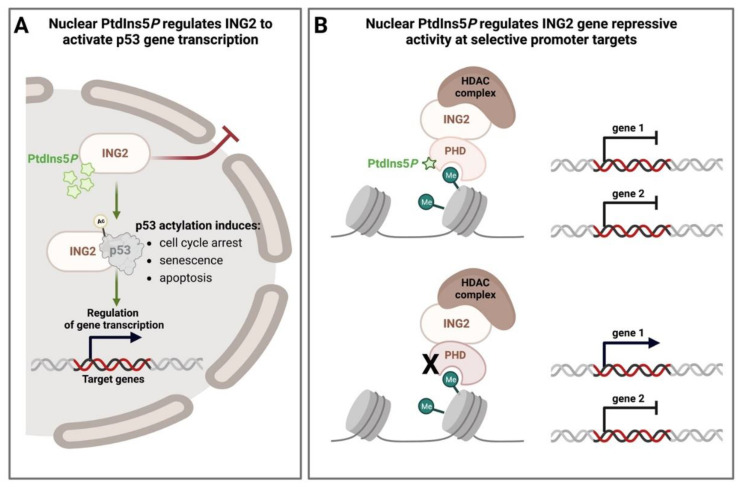
Schematic diagram representing the control of ING2 function by its interaction with nuclear PtdIns5*P*. (**A**) ING2 interacts with nuclear PtdIns5*P*, which promotes the retention of ING2 inside the nucleus, where it acetylases tumour suppressor p53. Acetylated p53 controls the activation or repression of target genes, and under stress conditions, it induces senescence, cell cycle arrest and apoptosis. The activity of PIP4K2β which controls the levels of nuclear PtdIns5*P* is reduced in response to cell stressors such as etoposide, which stimulates PIP4K2β phosphorylation by the p38-MAPK. (**B**) In response to stress signalling, such as by the treatment of etoposide, ING2 bound to PtdIns5*P* recruits the HDAC complex to specific promoter regions to cause gene repression (gene 1 and 2). In the absence of PtdIns5*P* binding, induced by ectopic expression of PIP4K2β, or by mutation of the PtdIns5*P* interaction site in the PHD finger of ING2, the recruitment of ING2 to selective promoters is reduced, preventing recruitment of the HDAC complex and gene repression. In this instance, gene 1 is no longer repressed, while gene 2 is still repressed.

PtdIns5*P* binds several nuclear proteins through plant homeodomains (PHD), which are conserved cross-braced zinc finger domains present predominantly in nuclear proteins and often mutated in human diseases [40,168,169]. PHD fingers also interact with methylated and non-methylated lysine residues in histone tails and regulate gene expression, in part by promoting the recruitment of co-transcriptional regulator complexes [170,171]. The first identified PHD finger binding to PtdIns5*P* was isolated from the inhibitor of growth protein 2 (ING2), a histone code reader and member of the ING family of tumour suppressors. INGs interact with and regulate the activity of the tumour suppressor p53, and regulate histone deacetylase (HDAC) and histone acetyltransferase (HAT) complexes [172] to regulate transcriptional output (Figure 3). The interaction between nuclear PtdIns5*P* and ING2 regulates ING2 function in at least two ways. PtdIns5*P* binding promotes ING2 localisation in the nucleus and helps ING2 to associate with discrete chromatin promoter targets [173]. PIP4K2β is a nuclear-localised lipid kinase that removes nuclear PtdIns5*P*. In conditions of stress, PIP4K2β is phosphorylated by the p38 MAP kinase, leading to a reduction in its activity and a consequent increase in nuclear PtdIns5*P* [105,173] (Figure 3B). Increased nuclear PtdIns5*P* increases nuclear localised ING2, which stimulates p53 acetylation to increase the expression of p53 target genes such as p21 [40,173] (Figure 3A). ING2 also strongly suppresses gene expression in response to cell stressors, such as etoposide, by recruiting a histone deacetylase complex to active promoters. Interestingly, blocking stress-induced PtdIns5*P*, or mutating ING2 such that it cannot bind PtdIns5*P* strongly, attenuates the repressive activity of ING2, but only at selective promoters [174] (Figure 3B). This is driven by changes in the recruitment of ING2 to these selective promotors rather than a change in its interaction with the HDAC complex. Thus, stress induced PtdIns5*P* controls the expression of genes required for cell cycle arrest, apoptosis and senescence [175,176].

**Figure 4:**
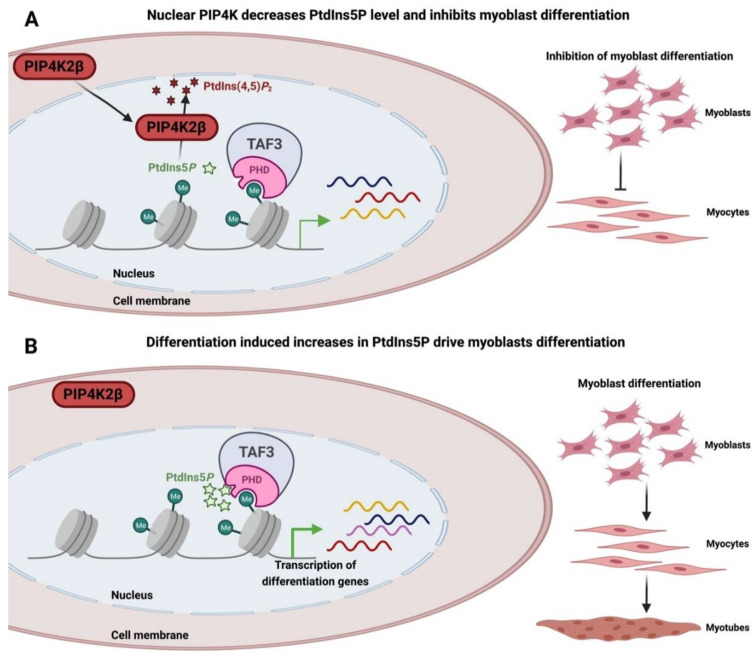
Schematic of the interaction between TAF3 and nuclear PtdIns5*P*, and how this modulates myogenic differentiation. (**A**) PIP4K2β is predominantly found in the nucleus where it phosphorylates PtdIns5*P* to PtdIns(4,5)*P*_2_, maintaining a low level of nuclear PtdIns5*P*. TAF3 bound to H3K4me3 does not induce myogenic differentiation under these conditions. (**B**) During differentiation, a myogenic signal induces PIP4K2β translocation into the cytoplasm, leading to a rise in nuclear PtdIns5*P*. PtdIns5*P* binds to the PHD finger of TAF3 and, in concert with binding to H3K4me3, drives the expression of genes required for myogenic differentiation. Mutations in the PHD finger that blocks interaction with PtdIns5*P* but maintain binding to H3K4me3 deregulate the expression of a subset of genes required for differentiation and phenocopy over expression of PIP4K2β which reduces nuclear PtdIns5*P*.

PtdIns5*P* also impacts on transcription through control of the basal transcription complex TFIID to impact on expression of a broad set of genes required for differentiation. TFIID binds to promoter regions of genes and helps to position RNA polymerase correctly for the start of transcription. In part, localisation of the complex to promoters is directed by one of its components, TAF3, which contains a PHD finger that interacts with H3K4me3. TAF3 is a critical factor that controls embryonic stem cell pluripotency, and is critical for myogenic differentiation [177,178,179]. The PHD finger of TAF3 also interacts with nuclear PPIns. Interaction of PtdIns5*P* with TAF3 controls the expression of a subset of TAF3-regulated genes, enabling changes in nuclear PtdIns5*P* to impact on myogenic differentiation [169]. Physiologically, under non-differentiating conditions, PIP4K2β, which is present in the nucleus, phosphorylates PtdIns5*P* to PtdIns(4,5)*P*_2,_ thereby attenuating the expression of genes required for myogenic differentiation induced by TAF3 (Figure 4A). Upon induction of differentiation, increased nuclear PtdIns5*P* is driven by differentiation-induced relocalisation of nuclear PIP4K2β into the cytoplasm (Figure 4B). TAF3 interaction with PtdIns5*P* and H3K4me3 drives expression of genes required for myogenic differentiation.

The molecular details of how interaction with PPIns regulates epigenetic signallers are still not clear, but are best understood in the role that PtdIns5*P* plays in the regulation of the ubiquitin-like PHD and RING finger domain-containing protein 1 (UHRF1). UHRF1 is a key integrator of epigenetic signalling, containing multiple reader domains and a ubiquitin writer domain. UHRF1 acts to safeguard the genome by maintaining global DNA methylation profiles, silencing repetitive elements, and protecting chromatin from DNA damaging agents [168]. PtdIns5*P* interacts with a polybasic region (PBR) in the C-terminus of UHRF1 to induce a conformational change and rearrangement of its domains, allowing the tandem Tudor domain (TTD) in the N-terminus to bind more strongly to H3K9me3 [168,180]. Although the exact consequences of PtdIns5*P* interaction with UHRF1 in vivo are not clear, UHRF1 is often upregulated in tumour cells, and targeting the allosteric PtdIns5*P* interaction site may have therapeutic value.

### 4.3. Nuclear PPIns and Regulation of Transcription Factors

While the above data illustrate how nuclear PPIns interact with and regulate epigenetic signalling, recent studies indicate that nuclear PPIns, in some instances, might play a more direct role in regulating transcription factor activity and stability. Steroidogenic factor-1 (SF-1) is a member of the nuclear receptor superfamily first identified as a regulator of p450 enzymes, and is now recognised as a global regulator of steroidogenic gene expression. SF-1 has essential roles in adrenogonadal development and differentiation [181]. Multiple factors modulate SF-1 activity, including post-translational modification, ligand binding and gene dosage. Structural analysis of SF-1 isolated from bacteria showed the presence of a large hydrophobic pocket occupied with the lipid phosphatidylglycerol (PG) [182,183].

**Figure 5:**
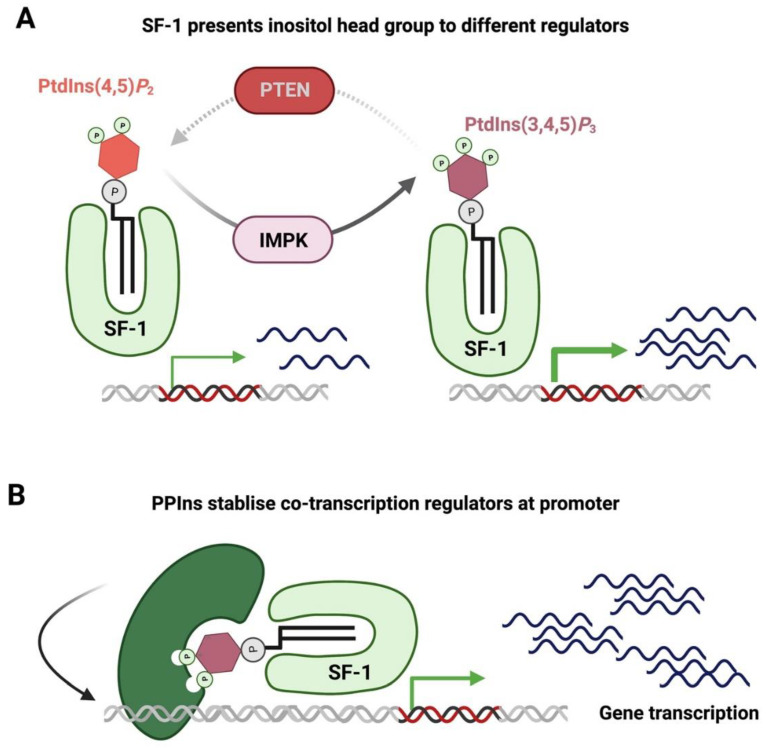
Schematic of how interaction of nuclear PPIns with SF-1 modulates transcriptional output. (**A**) SF-1 interacts with the fatty acyl tails of nuclear PtdIns(4,5)*P*_2_, exposing the inositol headgroup for further phosphorylation to PtdIns(3,4,5)*P*_3_ by the kinase IMPK. PtdIns(3,4,5)*P*_3_ bound to SF-1 is a better transcriptional activator than when it is bound to PtdIns(4,5)*P*_2_. PTEN is a 3-phosphatase that converts PtdIns(3,4,5)*P*_3_ back to PtdIns(4,5)*P*_2_, thereby attenuating transcriptional activation induced by IMPK. Interestingly, the PtdIns(4,5)*P*_2_ bound to SF-1 is not a good substrate for a classical class 1 PI3K, leading to the activation of gene transcription. (**B**) SF-1 binds a PPIns (in this case PtdIns(3,4,5)*P*_3_, but could potentially be any of the nuclear PPIns) and transports it to a genomic region such as a promoter. Here, the exposed head group acts as a recruiting platform to interact with and recruit co-transcriptional regulators that then impact on transcriptional output. It should be noted that SF-1 is a DNA sequence-specific binding transcription factor, and that targeting to genomic locations would require the presence of such a binding sequence. Whether SF-1 can move PPIns to any genomic site, perhaps through interaction with other transcription factors, is not clear.

**Figure 6:**
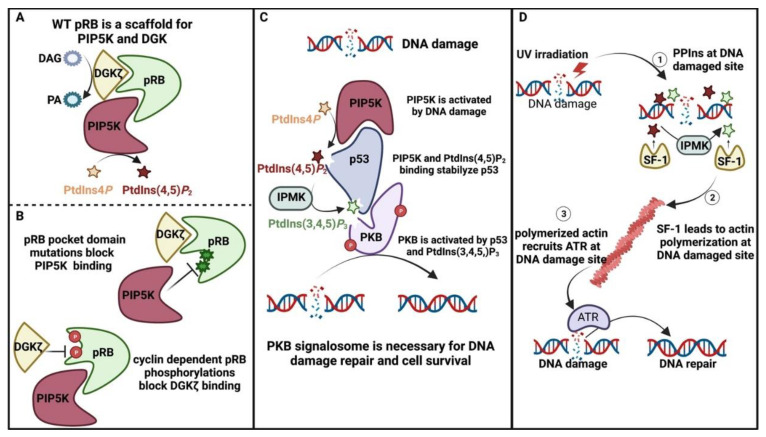
Schematic representing how PPIns function as protectors of the genome. (**A**,**B**) pRB acts to scaffold PIP5K and DGKζ. The interaction with PIP5K occurs through the small pocket domains, leads to activation of PIP5K, and mutations within this domain attenuate the interaction. DGKζ, instead, interacts with the c terminus of the large pocket domain, which also increases DGK activity. Cyclin-dependent phosphorylation of this site reduces interaction. (**C**) DNA damage induces the activation of PIP5K and an increase in PtdIns(4,5)*P*_2_ and PtdIns(3,4,5)*P*_3_ levels, which drive p53 stability and nuclear PKB activation. PKB activation facilitates DNA repair and cell survival. (**D**) DNA damage in response to UV irradiation leads to rapid SF-1-mediated PtdIns(4,5)*P*_2_ and PtdIns(3,4,5)*P*_3_ accumulation at sites of DNA damage. PtdIns(3,4,5)*P*_3_ is generated by IMPK. The increase in PPIns at sites of damage-induced actin polymerisation, which recruits ATR to the site of damage to facilitate DNA repair.

Further analysis showed that SF-1 has high affinity for PtdIns(4,5)*P*_2_ and PtdIns(3,4,5)*P*_3_ suggesting that, in mammalian cells, these might represent its natural ligands. Interestingly, SF-1 binds to PtdIns(4,5)*P*_2_ through the fatty acyl chains and can present the inositol head group for further phosphorylation. For example, SF-1 bound PtdIns(4,5)*P*_2_ can interact with, and be phosphorylated by, the inositol kinase, IMPK, to generate PtdIns(3,4,5)*P*_3_. Interestingly, SF-1 bound PtdIns(4,5)*P*_2_ is not a good substrate for a classical class 1 PI3K. Phosphorylation of PtdIns(4,5)*P*_2_ to PtdIns(3,4,5)*P*_3_ stimulates SF-1 transcriptional activity, and the loss of IMPK1 or overexpression of PTEN that removes PtdIns(3,4,5)*P*_3_ reduces the transcriptional regulation of SF-1 gene targets [114]. These studies suggest two important ideas. The first is that manipulation of a receptor-bound PPIn inositol head group can change downstream target regulation (Figure 5A), and the second is that receptors, such as SF-1, might facilitate localisation and presentation of PPIns at specific genomic regions such as a promoters where the exposed head group could interact with and recruit epigenetic regulators that then impact on transcription (Figure 5B). In fact, recent studies have shown PtdIns(3,4,5)*P*_3_ synthesis occurs at sites of DNA damage, which is essential for proper DNA repair. Interestingly, PtdIns(3,4,5)*P*_3_ production at the site of DNA damage appears to require both SF-1 and IMPK function (see Figure 6D) [184].

PtdIns(4,5)*P*_2_ is associated with the activation of transcription through a potential interaction with the RNA polymerase II (RNA Pol-II) transcription machinery [134,185]. Although it remains unclear whether there is a direct interaction between this PPIns and the RNA Pol-II itself, it has been found that PtdIns(4,5)*P*_2_ binds nuclear myosin 1 (NM1) at the nuclear lipid islets and regulates RNA Pol-II-dependent transcription [185]. NM1 is the largest member of the RNA Pol-II transcription machinery. The interaction of NM1 and PtdIns(4,5)*P*_2_ is required for NM1 to bind the rest of the transcription machinery complex and maintain active gene transcription. This is observed by the overexpression of a mutant NM1 that does not bind PtdIns(4,5)*P*_2_, which leads to a reduction in RNA Pol-II-mediated transcription (185]. PtdIns(4,5)*P*_2_ and PtdIns(3,4,5)*P*_3_ [186] have also been found in the nucleolus, a membrane-less self-aggregating nuclear body that controls ribosomal RNA (rRNA) synthesis by RNA-polymerase 1 (Pol1). PtdIns(4,5)*P*_2_ has been immunolocalised to this compartment and can interact directly with Pol1, fibrillarin and UBF (upstream binding factor), and inhibition of PtdIns(4,5)*P*_2_ synthesis in the nucleolus reduces rRNA synthesis [134]. PtdIns(3,4,5)*P*_3_ synthesis in the nucleolus is controlled in part by the presence and activation of class 1 PI3Kβ, which drives 47s rRNA synthesis. Upregulation of this PtdIns(3,4,5)*P*_3_ pathway might be important in the development of endometrial tumours [134].

### 4.4. Nuclear PPIns and Their Role in Protecting the Genome

The maintenance of the integrity of genetic material is a prerequisite for healthy organismal living and for the generation of healthy progeny. Each cell experiences thousands of DNA lesions per day, threatening cellular and organismal viability. Multiple factors, both internal and external, such as reactive oxygen species or sunlight, can damage our DNA, which, if not repaired properly, can lead to cell dysfunction and, ultimately, cell death. Moreover, the accumulation of DNA mutations is a major factor associated with loss of stem cell function, reduced regenerative capacity, increased ageing and eventual organismal death. Two tumour suppressor proteins, retinoblastoma (pRB) [187] and p53 [188], are critical for controlling how cells respond to excessive cell proliferative signals, such as those induced by oncogenes, and to DNA damage. Under adverse conditions, pRB and p53 stop cell proliferation, induce repair to any damage and, if repair is unsuccessful, induce cellular apoptosis. Not surprisingly, these proteins are often mutated, deleted or targeted for inhibition during tumour development and viral infection. pRB contains a pocket domain which is essential for its interaction with the E2F transcription factors and other pRB binding proteins, and this site is often mutated in human tumours or is the target for viral proteins that block pRB function. In a screen to identify novel pRB binding proteins PIP5K, which synthesises PtdIns(4,5)*P*_2_, was found to specifically interact with the pocket domain of pRB and disease mutations within this domain, or viral proteins that block pRB function strongly reduced the interaction with PIP5K. pRB binding to PIP5K increased PIP5K activity in vitro. Expression of large T antigen to block the pRB/PIP5K interaction strongly reduced nuclear PtdIns(4,5)*P*_2_ levels in vivo. These data were the first to suggest that a bona fide tumour suppressor and master cell cycle regulator could interact with, and regulate the levels of, nuclear PPIns [189]. Further studies showed that pRB also interacts specifically with DGKζ at a site different from its interaction with PIP5K. The pRB/DGKζ interaction is strongly regulated by the phosphorylation of RB during the cell cycle. Furthermore, DGKζ interaction with pRB is required for efficient survival after DNA damage induced by gamma irradiation [190,191]. These data suggest that pRB might act as a scaffold protein to integrate PPIns signalling in the nucleus (Figure 6A,B).

Later studies showed that PIP5K also interacts with the p53 tumour suppressor protein and that knockdown of PIP5K1A, or inhibition of its enzymatic activity, strongly reduced the post-transcriptional expression of p53 [192]. Surprisingly, p53 was found to directly interact with PtdIns(4,5)*P*_2_, which induced an interaction between p53 and the heat shock protein HSP27, leading to p53 stabilisation. Further studies have revealed that PtdIns(4,5)*P*_2_ bound to p53 is a substrate for IMPK, which generates p53 bound to PtdIns(3,4,5)*P*_3_. This complex appears to generate a PKB signalosome, which leads to the activation of nuclear PKB. The activation of nuclear PKB appears to be required for proper DNA repair and cell survival in response to a DNA damage signal [193] (Figure 6C). These studies may resolve a long-standing issue in the PKB field as to how and where nuclear PKB might become activated. PKB is one of the most well-studied targets of PtdIns(3,4,5)*P*_3_ [194,195,196], with the interaction occurring through the PH domain of PKB. Nuclear PKB associates with nucleophosmin (NPM/B23) to regulate the expression of genes involved in cell survival [36]. Biochemical assays found that nucleophosmin is also a nuclear target of PtdIns(3,4,5)*P*_3_, and that the interaction of B23 with PtdIns(3,4,5)*P*_3_ is required for interaction with PKB in the nucleus.

Recent studies have also implicated a more direct role for nuclear PPIns in the control of DNA damage responses. In response to UV irradiation, DNA breaks are sensed by the MRN complex [197], which leads to the recruitment of the DNA damage-regulated PI3K-like protein kinases ATM, ATR and DNA-PK. These kinases phosphorylate a plethora of downstream targets that impact on DNA repair, cell cycle progression, RNA splicing and cell survival [198]. How exactly the whole process of DNA repair is coordinated is not completely clear. UV damage and other DNA damage or cell stressors induce changes in nuclear PPIns [173,193,199]. In this study, the authors used nuclear-targeted PH domains that bind specific PPIns as agents to sequester these PPIns. Overexpression of specific domains that bind to either PtdIns(4,5)*P*_2_ or PtdIns(3,4,5)*P*_3_ blocked the accumulation of ATR, but not ATM or DNA-PK, at sites of DNA damage, in a manner that depended on IMPK (production of PtdIns(3,4,5)*P*_3_) and on nuclear actin polymerisation. These data suggest that rapid PPIns signalling in response to DNA damage controls specific DNA damage signalling that impacts on DNA repair and cell survival (Figure 6D).

DNA is also susceptible to oxidative damage, which can occur in response to damage to mitochondria or increased cell metabolism. Cells have developed multiple mechanisms to sense increased oxidative stress, which eventually impact on the expression of genes that bestow enhanced adaptive cell capabilities for dealing with subsequent exposures to oxidative stress. During oxidative stress, PIP4K2β, which controls nuclear PtdIns5*P*, is phosphorylated on two residues, threonine 322 and serine 326 [173]. Phosphorylation of these residues induces their interaction with the phosphospecific-prolyl-isomerase Pin1, leading to a Pin1 activity-dependent decrease in nuclear PIP4K2β activity. The subsequent increase in nuclear PtdIns5*P* drives increased expression of genes involved in oxidative stress adaptation. Knockout of Pin1, or overexpression of PIP4K, reduces stress-induced PtdIns5*P* accumulation, attenuates the increased expression of these genes and compromises cell survival in response to oxidative damage [200]. How exactly PtdIns5*P* impacts on these genes is not clear, but may involve regulation of NRF2/Keap1, as many of the genes regulated by PtdIns5*P* are downstream targets of this pathway. In this respect, PtdIns5*P* has been linked to the regulation of the serine/threonine protein kinase B (PKB)/AKT, which plays an important role in stress adaptive responses, through regulation of the NRF2/Keap1 pathway. PKB is phosphorylated and activated in response to DNA damage caused by H_2_O_2_ in a manner dependent on PtdIns5*P* and overexpression of PIP4K2α to remove PtdIns5*P*, attenuated PKB activation [199].

### 4.5. Nuclear Speckle and mRNA Machinery

The presence of PtdIns(4,5)*P*_2_ at the nuclear speckles has been shown using co-staining assays with nuclear speckles proteins, including the splicing factor SRSF2 and the speckle protein SON [61,62]. While PIP4K can synthesise PtdIns(4,5)*P*_2_, PIP5Ks are likely to be the major synthetic enzyme for the production of nuclear PtdIns(4,5)*P*_2_. In contrast to PIP5Ks, PIP4Ks are not as abundant, and their physiological functions have to be fully revealed. PIP4K2β has been found in the nucleus at nuclear speckles [68]. PIP4K2β interacts with the nuclear protein SPOP (speckle-type POZ domain protein) [201]. SPOP is a nuclear speckle-associated BTB (Broad complex/Tramtrack/brick-a-brac) domain-containing protein that functions as a substrate adaptor of the E3 ubiquitin ligases Cul3 [202,203,204]. SPOP and PIP4K2β interact in vitro and in vivo, and they are both found at the nuclear speckles. The Cul3-SPOP complex regulates the ubiquitylation of PIP4K2β, among other proteins; these ubiquitylation processes seem to be dependent on, and regulated through, the MKK6-p38 MAPK (MapKinase) pathway. Interestingly, the substrate of PIP4K2β, PtdIns5*P* activates the ubiquitin ligase activity of the Cul3-SPOP complex through p38-MAPK signalling [201].

Nuclear speckle PtdIns(4,5)*P*_2_ is detergent-resistant, and the morphology and density of these membrane-less structures are cell-cycle dependent [61]. As described in “The nucleus and nuclear PPIn transport” section, nuclear speckles are the hub for splicing and pre-mRNA processing machinery. Interestingly, immuno-depletion of PtdIns(4,5)*P*_2_ leads to the inhibition of splicing in vitro, strengthening the importance of PtdIns(4,5)*P*_2_ in regulating splicing events [61]. Moreover, PtdIns(4,5)*P*_2_ co-immunoprecipitates with associated members of the splicing and pre-mRNA complexes. Although the clear role of PtdIns(4,5)*P*_2_ in splicing and pre-mRNA processing remains to be determined, these findings suggest that PtdIns(4,5)*P*_2_ is an important member and potential regulator of these nuclear processes. PtdIns(4,5)*P*_2_ and PtdIns(3,4,5)*P*_3_ have also been linked to the nuclear speckle protein ALY/REF [205]. ALY/REF is a nuclear factor protein that regulates the nuclear export of mature mRNA to the cytoplasm. The interaction of PtdIns(4,5)*P*_2_ and PtdIns(3,4,5)*P*_3_ with the N-terminus of ALY/REF regulates its nuclear speckle localisation, which directly impacts its mRNA export function. In addition, ALY/REF has been associated with PI3K and its main protein target AKT [205]. PI3K-dependent nuclear translocation of AKT allows phosphorylation of ALY/REF, which is important for cell proliferation and mRNA export. Disruption of ALY/REF phosphorylation by AKT activity leads to a significant decrease in cell growth, proliferation and mRNA export [205]. Specific mRNA expression is also regulated by the interaction of PIP5KIα and the product of its kinase activity, PtdIns(4,5)*P*_2_, with the nuclear poly(A) polymerase Star-PAP [206]. Star-PAP is directly phosphorylated by casein kinase Iα (CKIα) through PtdIns(4,5)*P*_2_-dependent protein kinase activity [207], and star-PAP activity in vitro is strongly increased by PtdIns(4,5)*P*_2_. The interaction of PIP5KIα and CKIα with star-PAP regulates the expression of a subgroup of star-PAP target mRNAs by controlling its association with selective mRNA.

## 5. Concluding Notes

In the early 1980s, key discoveries in nuclear PPIns centred around biochemical studies aimed at ensuring that the nuclear pool of PPIns was not a consequence of contamination from the much larger pool in the cytoplasm (plasma membrane, ER, Golgi) and, subsequently, on demonstrating that the nuclear pool could be regulated distinctly from other pools of PPIns. Further studies began to define nuclear targets that potentially interact with nuclear pools of PPIns, which effectively define downstream signalling pathways. In fact, we now have a plethora of signalling proteins that are nuclear, bind PPIns and potentially impact on all aspects of nuclear functions, though we lack considerable knowledge of how these pathways are controlled and coordinated. We still do not understand how the nuclear pool of PPIns is established, how it is maintained and how it is controlled and manipulated. The first two are rather perplexing, given that the immuno-localisation studies for PtdIns(4,5)*P*_2_ suggest that it is highly localised in membrane-less nuclear bodies, such as splicing speckles, islets and the nucleolus. This begs some simple questions. How do PPIns enter the nucleus and, once there, how are membrane-loving lipids maintained in membrane-less compartments or at specific genomic regions? PITP and or SF-1 may provide novel mechanisms to transport PPIns to specific genomic regions, such as promoters, where the exposed inositol head group can be presented to regulate epigenetic signalling. Alternatively, the regulation of subsets of genes by PPIns may occur through selective localisation of genes next to PPIns-rich regions of the nucleus. Proximity-based TSA assays have illustrated how genes that are upregulated often become more closely associated with nuclear splicing speckles where PtdIns(4,5)*P*_2_ is localized [208]. Finally, how enzymes that modulate nuclear PPIns are controlled is not well understood, which is critical, as this underpins how environmental cues (growth factors, DNA damage, etc.) impact on the levels of nuclear PPIns pools. In part, this lack of knowledge is driven by the lack of nuclear-specific isoforms that only regulate nuclear pools of PPIns. In most instances, the enzymes shuttle between the two compartments and control their cognate lipid in both compartments. For example, PIP4K2β has a nuclear localisation sequence that allows it to shuttle between compartments [106] and is phosphorylated in response to activation of the p38-MAPK stress pathway [173]. PIP5K1α also shuttles between the cytoplasm and nucleus. PIP5K1α is sumoylated at three different lysine residues, and while sumoylation at lysine 244 controls nuclear entry, sumoylation at lysine 490 controls subnuclear localisation to the nucleolus. Sumoylation at Lys-490 induces association of PIP5K1α with the chromatin silencing machinery, including heterochromatin protein 1α (HP1α) and the epigenetic histone modification H3K9me3, to inhibit the expression of target ribosomal DNA (rDNA). Phospholipase Cβ1, which hydrolyses nuclear PtdIns(4,5)*P*_2_ to generate DAG and nuclear Ins(1,4,5)*P*_3_ shuttles between the cytoplasm and the nucleus. Of the two known isoforms of PLCβ1 (1A and 1B), PLCβ1B was suggested to be more localised to the nucleus compared to PLCβ1A, due to the presence of a nuclear export sequence in an alternatively spliced exon in PLCβ1A. PLCβ1 is phosphorylated by the P42-MAPK, which leads to an increase in its nuclear activity [209] and subsequent phosphorylation by PKC acts to switch off PLCβ1 [210]. Analysing the activity of these enzymes in vivo is even more challenging, given the lack of tools to study phosphoinositide dynamics in real-time in the nucleus. Clearly, we need a much more detailed map of how nuclear PPIns are modulated distinctly to the cytoplasmic pool if specific targeting of the nuclear PPIn pathway is to be used for therapeutic benefit.

## Data Availability

Not applicable.

## References

[1] Balla T., Szentpetery Z., Kim Y.J., Dingjan I., Linders P.T.A., Verboogen D.R.J., Revelo N.H., ter Beest M., Bogaart G.v.D., Sbrissa D. (2009). Phosphoinositide Signaling: New Tools and Insights. Physiology.

[2] Balla T. (2013). Phosphoinositides: Tiny Lipids With Giant Impact on Cell Regulation. Physiol. Rev..

[3] Morelec-Coulon M.J., Faure M. (1958). Glycerol-inositol-phosphatidic acid. IV. Molecular structure. Bull. Soc. Chim. Biol..

[4] Hawthorne J.N. (1960). The inositol phospholipids. J. Lipid Res..

[5] Hawthorne J.N., Kemp P., Ellis R.B. (1960). Phosphoinositides. 2. The inositol 1-phosphate structure in liver phosphatidylinositol. Biochem. J..

[6] Irvine R.F. (2003). Nuclear lipid signalling. Nat. Rev. Mol. Cell Biol..

[7] Lemmon M.A. (2003). Phosphoinositide Recognition Domains. Traffic.

[8] Lemmon M. (2007). Pleckstrin homology (PH) domains and phosphoinositides. Biochem. Soc. Symp..

[9] Hammond G.R., Balla T. (2015). Polyphosphoinositide binding domains: Key to inositol lipid biology. Biochim. Biophys. Acta (BBA)—Mol. Cell Biol. Lipids.

[10] Ferguson K.M., Lemmon M.A., Schlessinger J., Sigler P.B. (1995). Structure of the high affinity complex of inositol trisphosphate with a phospholipase C pleckstrin homology domain. Cell.

[11] Harlan J.E., Hajduk P.J., Yoon H.S., Fesik S.W. (1994). Pleckstrin homology domains bind to phosphatidylinositol-4,5-bisphosphate. Nature.

[12] Singh N., Reyes-Ordoñez A., Compagnone M.A., Moreno J.F., Leslie B.J., Ha T., Chen J. (2021). Redefining the specificity of phosphoinositide-binding by human PH domain-containing proteins. Nat. Commun..

[13] Stauffer T.P., Ahn S., Meyer T. (1998). Receptor-induced transient reduction in plasma membrane PtdIns(4,5)P2 concentration monitored in living cells. Curr. Biol..

[14] Guillou H., Lécureuil C., Anderson K.A., Suire S., Ferguson G.J., Ellson C.D., Gray A., Divecha N., Hawkins P.T., Stephens L.R. (2007). Use of the GRP1 PH domain as a tool to measure the relative levels of PtdIns(3,4,5)P3 through a protein-lipid overlay approach. J. Lipid Res..

[15] Clark J., Anderson K.E., Juvin V., Smith T.S., Karpe F., Wakelam M.J.O., Stephens L.R., Hawkins P.T. (2011). Quantification of PtdInsP3 molecular species in cells and tissues by mass spectrometry. Nat. Methods.

[16] Milne S.B., Ivanova P.T., DeCamp D., Hsueh R.C., Brown H.A. (2005). A targeted mass spectrometric analysis of phosphatidylinositol phosphate species. J. Lipid Res..

[17] Vadnal R.E., Parthasarathy R. (1989). The identification of a novel inositol lipid, phosphatidylinostiol trisphosphate (PIP3), in rat cerebrum using in vivo techniques. Biochem. Biophys. Res. Commun..

[18] Lands W.E. (1960). Metabolism of glycerolipids. 2. The enzymatic acylation of lysolecithin. J. Biol. Chem..

[19] Murphy R.C., Folco G. (2019). Lysophospholipid acyltransferases and leukotriene biosynthesis: Intersection of the Lands cycle and the arachidonate PI cycle. J. Lipid Res..

[20] Anderson K.E., Kielkowska A., Durrant T.N., Juvin V., Clark J., Stephens L.R., Hawkins P.T. (2013). Lysophosphatidylinositol-Acyltransferase-1 (LPIAT1) Is Required to Maintain Physiological Levels of PtdIns and PtdInsP2 in the Mouse. PLoS ONE.

[21] Barneda D., Cosulich S., Stephens L., Hawkins P. (2019). How is the acyl chain composition of phosphoinositides created and does it matter?. Biochem. Soc. Trans..

[22] Patki V., Virbasius J., Lane W.S., Toh B.-H., Shpetner H.S., Corvera S. (1997). Identification of an early endosomal protein regulated by phosphatidylinositol 3-kinase. Proc. Natl. Acad. Sci. USA.

[23] Fiume R., Keune W.J., Faenza I., Bultsma Y., Ramazzotti G., Jones D.R., Martelli A.M., Somner L., Follo M.Y., Divecha N. (2012). Nuclear phosphoinositides: Location, regulation and function. Phosphoinosit. II Divers. Biol. Funct..

[24] Di Paolo P.G., De C.P. (2006). Phosphoinositides in cell regulation and membrane dynamics. Nature.

[25] Giuriato S., Blero D., Robaye B., Bruyns C., Payrastre B., Erneux C. (2002). SHIP2 overexpression strongly reduces the proliferation rate of K562 erythroleukemia cell line. Biochem. Biophys. Res. Commun..

[26] Clément S., Krause U., Desmedt F., Tanti J.-F., Behrends J., Pesesse X., Sasaki T., Penninger J., Doherty M., Malaisse W. (2001). The lipid phosphatase SHIP2 controls insulin sensitivity. Nature.

[27] Luo J.-M., Yoshida H., Komura S., Ohishi N., Pan L., Shigeno K., Hanamura I., Miura K., Iida S., Ueda R. (2003). Possible dominant-negative mutation of the SHIP gene in acute myeloid leukemia. Leukemia.

[28] Marion E., Kaisaki P.J., Pouillon V., Gueydan C., Levy J.C., Bodson A., Krzentowski G., Daubresse J.-C., Mockel J., Behrends J. (2002). The Gene INPPL1, Encoding the Lipid Phosphatase SHIP2, Is a Candidate for Type 2 Diabetes In Rat and Man. Diabetes.

[29] Halstead J.R., Jalink K., Divecha N. (2005). An emerging role for PtdIns(4,5)P2-mediated signalling in human disease. Trends Pharmacol. Sci..

[30] McCrea H.J., De Camilli P., Inoue K., Ishibe S., Silswal N., Parelkar N.K., Wacker M.J., Brotto M., Andresen J. (2009). Mutations in Phosphoinositide Metabolizing Enzymes and Human Disease. Physiology.

[31] Carrat G.R., Haythorne E., Tomas A., Haataja L., Müller A., Arvan P., Piunti A., Cheng K., Huang M., Pullen T.J. (2020). The type 2 diabetes gene product STARD10 is a phosphoinositide-binding protein that controls insulin secretory granule biogenesis. Mol. Metab..

[32] Bridges D., Saltiel A.R. (2012). Phosphoinositides in Insulin Action and Diabetes. Current Topics in Microbiology and Immunology.

[33] Kavran J.M., Klein D.E., Lee A., Falasca M., Isakoff S.J., Skolnik E.Y., Lemmon M.A. (1998). Specificity and Promiscuity in Phosphoinositide Binding by Pleckstrin Homology Domains. J. Biol. Chem..

[34] Paterson H.F., Savopoulos J.W., Perisic O., Cheung R., Ellis M.V., Williams R.L., Katan M. (1995). Phospholipase C *δ*1 requires a pleckstrin homology domain for interaction with the plasma membrane. Biochem. J..

[35] Yagisawa H., Sakuma K., Paterson H.F., Cheung R., Allen V., Hirata H., Watanabe Y., Hirata M., Williams R.L., Katan M. (1998). Replacements of single basic amino acids in the pleckstrin homology domain of phospholipase C-delta1 alter the ligand binding, phospholipase activity, and interaction with the plasma membrane. J. Biol. Chem..

[36] Kwon I.-S., Lee K.-H., Choi J.W., Ahn J.-Y. (2010). PI(3,4,5)P3 regulates the interaction between Akt and B23 in the nucleus. BMB Rep..

[37] Kanai F., Liu H., Field S., Akbary H., Matsuo T., Brown G.E., Cantley L., Yaffe M.B. (2001). The PX domains of p47phox and p40phox bind to lipid products of PI(3)K. Nature.

[38] Ellson C.D., Andrews S., Stephens L.R., Hawkins P.T. (2002). The PX domain: A new phosphoinositide-binding module. J. Cell Sci..

[39] Chandra M., Chin Y.K.-Y., Mas C., Feathers J.R., Paul B., Datta S., Chen K.-E., Jia X., Yang Z., Norwood S.J. (2019). Classification of the human phox homology (PX) domains based on their phosphoinositide binding specificities. Nat. Commun..

[40] Gozani O., Karuman P., Jones D.R., Ivanov D., Cha J., Lugovskoy A.A., Baird C.L., Zhu H., Field S.J., Lessnick S.L. (2003). The PHD Finger of the Chromatin-Associated Protein ING2 Functions as a Nuclear Phosphoinositide Receptor. Cell.

[41] Kaadige M.R., Ayer D.E. (2006). The Polybasic Region That Follows the Plant Homeodomain Zinc Finger 1 of Pf1 Is Necessary and Sufficient for Specific Phosphoinositide Binding. J. Biol. Chem..

[42] Stenmark H., Aasland R., Driscoll P. (2002). The phosphatidylinositol 3-phosphate-binding FYVE finger. FEBS Lett..

[43] Itoh T., Koshiba S., Kigawa T., Kikuchi A., Yokoyama S., Takenawa T. (2001). Role of the ENTH domain in phosphatidylinositol-4,5-bisphosphate binding and endocytosis. Science.

[44] Ford M.G., Pearse B.M., Higgins M.K., Vallis Y., Owen D.J., Gibson A., Hopkins C.R., Evans P.R., McMahon H.T. (2001). Simultaneous binding of PtdIns(4,5)P2 and clathrin by AP180 in the nucleation of clathrin lattices on membranes. Science.

[45] Lewis A.E., Sommer L., Arntzen M., Strahm Y., Morrice N.A., Divecha N., D’Santos C.S. (2011). Identification of Nuclear Phosphatidylinositol 4,5-Bisphosphate-Interacting Proteins by Neomycin Extraction. Mol. Cell. Proteom..

[46] Brown D.A. (2015). PIP2Clustering: From model membranes to cells. Chem. Phys. Lipids.

[47] Sztacho M., Šalovská B., Červenka J., Balaban C., Hoboth P., Hozák P. (2021). Limited Proteolysis-Coupled Mass Spectrometry Identifies Phosphatidylinositol 4,5-Bisphosphate Effectors in Human Nuclear Proteome. Cells.

[48] Santagata S., Boggon T.J., Baird C.L., Gomez C.A., Zhao J., Shan W.S., Myszka D.G., Shapiro L. (2001). G-Protein Signaling Through Tubby Proteins. Science.

[49] Quinn K.V., Behe P., Tinker A. (2008). Monitoring changes in membrane phosphatidylinositol 4,5-bisphosphate in living cells using a domain from the transcription factor tubby. J. Physiol..

[50] Doucet C.M., Hetzer M.W. (2010). Nuclear pore biogenesis into an intact nuclear envelope. Chromosoma.

[51] la Cour T., Kiemer L., Mølgaard A., Gupta R., Skriver K., Brunak S. (2004). Analysis and prediction of leucine-rich nuclear export signals. Protein Eng. Des. Sel..

[52] Cokol M., Nair R., Rost B. (2000). Finding nuclear localization signals. EMBO Rep..

[53] Holmer L., Worman H. (2001). Inner nuclear membrane proteins: Functions and targeting. Cell. Mol. Life Sci..

[54] Misteli T. (2007). Beyond the Sequence: Cellular Organization of Genome Function. Cell.

[55] Carrero G., Hendzel M., de Vries G. (2006). Modelling the compartmentalization of splicing factors. J. Theor. Biol..

[56] Cremer T., Cremer C. (2001). Chromosome territories, nuclear architecture and gene regulation in mammalian cells. Nat. Rev. Genet..

[57] Ho L., Crabtree G.R. (2010). Chromatin remodelling during development. Nature.

[58] Clapier C.R., Iwasa J., Cairns B.R., Peterson C.L. (2017). Mechanisms of action and regulation of ATP-dependent chromatin-remodelling complexes. Nat. Rev. Mol. Cell Biol..

[59] Spector D., Lamond A. (2011). Nuclear Speckles. Cold Spring Harb. Perspect. Biol..

[60] Cremer T., Kreth G., Koester H., Fink R.H.A., Heintzmann R., Cremer M., Solovei I., Zink D., Cremer C. (2000). Chromosome Territories, Interchromatin Domain Compartment, and Nuclear Matrix: An Integrated View of the Functional Nuclear Architecture. Crit. Rev. Eukaryot. Gene Expr..

[61] Osborne S., Thomas C., Gschmeissner S., Schiavo G. (2001). Nuclear PtdIns(4,5)P2 assembles in a mitotically regulated particle involved in pre-mRNA splicing. J. Cell Sci..

[62] Kalasova I., Fáberová V., Kalendová A., Yildirim S., Uličná L., Venit T., Hozák P. (2016). Tools for visualization of phosphoinositides in the cell nucleus. Histochem. Cell Biol..

[63] Divecha N., Banfić H., Irvine R.F. (1993). Inositides and the nucleus and inositides in the nucleus. Cell.

[64] Divecha N., Banfic H., Irvine R.F. (1991). The polyphosphoinositide cycle exists in the nuclei of Swiss 3T3 cells under the control of a receptor (for IGF-1) in the plasma membrane, and stimulation of the cycle increases nuclear diacylglycerol and apparently induces translocation of protein kinase. EMBO J..

[65] Banfić H., Žižak M., Divecha N., Irvine R.F. (1993). Nuclear diacylglycerol is increased during cell proliferation in vivo. Biochem. J..

[66] Vann L.R., Wooding P.F., Irvine R.F., Divecha N. (1997). Metabolism and possible compartmentalization of inositol lipids in isolated rat-liver nuclei. Biochem. J..

[67] Cocco L., Gilmour R.S., Ognibene A., Letcher A.J., Manzoli F.A., Irvine R.F. (1987). Synthesis of polyphosphoinositides in nuclei of Friend cells. Evidence for polyphosphoinositide metabolism inside the nucleus which changes with cell differentiation. Biochem. J..

[68] Boronenkov I.V., Loijens J.C., Umeda M., Anderson R.A. (1998). Phosphoinositide signaling pathways in nuclei are associated with nuclear speckles containing pre-mRNA processing factors. Mol. Biol. Cell.

[69] De Vries K.J., Heinrichs A.A.J., Cunningham E., Brunink F., Westerman J., Somerharju P.J., Cockcroft S., Wirtz K.W.A., Snoek G.T. (1995). An isoform of the phosphatidylinositol-transfer protein transfers sphingomyelin and is associated with the Golgi system. Biochem. J..

[70] Rubbini S., Cocco L., Manzoli L., Lutterman J., Billi A., Matteucci A., Wirtz K. (1997). Phosphoinositide Signalling in Nuclei of Friend Cells: DMSO-Induced Differentiation Reduces the Association of Phosphatidylinositol-Transfer Protein with the Nucleus. Biochem. Biophys. Res. Commun..

[71] de Vries K., Westerman J., Bastiaens P., Jovin T., Wirtz K., Snoek G. (1996). Fluorescently Labeled Phosphatidylinositol Transfer Protein Isoforms (α and β), Microinjected into Fetal Bovine Heart Endothelial Cells, Are Targeted to Distinct Intracellular Sites. Exp. Cell Res..

[72] Tribble E.K., Ivanova P.T., Grabon A., Alb J.G., Faenza I., Cocco L., Brown H.A., Bankaitis V.A. (2016). Quantitative profiling of the endonuclear glycerophospholipidome of murine embryonic fibroblasts. J. Lipid Res..

[73] Carrillo N.D., Chen M., Cryns V.L., Anderson R.A. (2023). Lipid transfer proteins initiate nuclear phosphoinositide signaling. bioRxiv.

[74] Prasanth K.V., Sacco-Bubulya P.A., Prasanth S.G., Spector D.L. (2003). Sequential Entry of Components of Gene Expression Machinery into Daughter Nuclei. Mol. Biol. Cell.

[75] Sobol M., Yildirim S., Philimonenko V.V., Marášek P., Castaño E., Hozák P. (2013). UBF complexes with phosphatidylinositol 4,5-bisphosphate in nucleolar organizer regions regardless of ongoing RNA polymerase I activity. Nucleus.

[76] Smith C.D., Wells W.W. (1983). Phosphorylation of rat liver nuclear envelopes. I. Characterization of in vitro protein phosphorylation. J. Biol. Chem..

[77] Shah Z.H., Jones D.R., Sommer L., Foulger R., Bultsma Y., D’Santos C., Divecha N. (2013). Nuclear phosphoinositides and their impact on nuclear functions. FEBS J..

[78] Gaullier J.-M., Simonsen A., D’Arrigo A., Bremnes B., Stenmark H., Aasland R. (1998). FYVE fingers bind PtdIns(3)P. Nature.

[79] Stenmark H., Aasland R. (1999). FYVE-finger proteins--effectors of an inositol lipid. J. Cell Sci..

[80] Gillooly D.J., Morrow I.C., Lindsay M., Gould R., Bryant N.J., Gaullier J., Parton R.G., Stenmark H. (2000). Localization of phosphatidylinositol 3-phosphate in yeast and mammalian cells. EMBO J..

[81] Norris F.A., Majerus P.W. (1994). Hydrolysis of phosphatidylinositol 3,4-bisphosphate by inositol polyphosphate 4-phosphatase isolated by affinity elution chromatography. J. Biol. Chem..

[82] Norris F.A., Auethavekiat V., Majerus P.W. (1995). The Isolation and Characterization of cDNA Encoding Human and Rat Brain Inositol Polyphosphate 4-Phosphatase. J. Biol. Chem..

[83] Devereaux K., Dall’armi C., Alcazar-Roman A., Ogasawara Y., Zhou X., Wang F., Yamamoto A., De Camilli P., Di Paolo G. (2013). Regulation of Mammalian Autophagy by Class II and III PI 3-Kinases through PI3P Synthesis. PLoS ONE.

[84] Franco I., Gulluni F., Campa C.C., Costa C., Margaria J.P., Ciraolo E., Martini M., Monteyne D., De Luca E., Germena G. (2014). PI3K Class II α Controls Spatially Restricted Endosomal PtdIns3P and Rab11 Activation to Promote Primary Cilium Function. Dev. Cell.

[85] Gulluni F., De Santis M.C., Margaria J.P., Martini M., Hirsch E. (2019). Class II PI3K Functions in Cell Biology and Disease. Trends Cell Biol..

[86] Didichenko S.A., Thelen M. (2001). Phosphatidylinositol 3-Kinase C2α Contains a Nuclear Localization Sequence and Associates with Nuclear Speckles. J. Biol. Chem..

[87] Višnjić D., Crljen V., Ćurić J., Batinić D., Volinia S., Banfić H. (2002). The activation of nuclear phosphoinositide 3-kinase C2β in all-*trans*-retinoic acid-differentiated HL-60 cells. FEBS Lett..

[88] Banfic H., Visnjic D., Mise N., Balakrishnan S., Deplano S., Korchev Y.E., Domin J. (2009). Epidermal growth factor stimulates translocation of the class II phosphoinositide 3-kinase PI3K-C2β to the nucleus. Biochem. J..

[89] Huang P.T., Einav S., Asquith C.R.M. (2021). PIKfyve: A lipid kinase target for COVID-19, cancer and neurodegenerative disorders. Nat. Rev. Drug Discov..

[90] Clague M.J., Lorenzo Ó. (2005). The Myotubularin Family of Lipid Phosphatases. Traffic.

[91] Robinson F.L., Dixon J.E. (2006). Myotubularin phosphatases: Policing 3-phosphoinositides. Trends Cell Biol..

[92] Divecha N., Letcher A.J., Banfic H.H., Rhee S.G., Irvine R.F. (1995). Changes in the components of a nuclear inositide cycle during differentiation in murine erythroleukaemia cells. Biochem. J..

[93] Fáberová V., Kalasová I., Krausová A., Hozák P. (2020). Super-Resolution Localisation of Nuclear PI(4)P and Identification of Its Interacting Proteome. Cells.

[94] Mao Y., Manford A., Xia T., Saxena A., Stefan C., Hu F., Emr S. (2010). Crystal structure of the Yeast Sac1: Implications for its phosphoinositide phosphatase function. EMBO J..

[95] Zewe J.P., Wills R.C., Sangappa S., Goulden B.D., Hammond G.R. (2018). SAC1 degrades its lipid substrate PtdIns4P in the endoplasmic reticulum to maintain a steep chemical gradient with donor membranes. eLife.

[96] Clarke J.H., Letcher A.J., D’Santos C.S., Halstead J.R., Irvine R.F., Divecha N. (2001). Inositol lipids are regulated during cell cycle progression in the nuclei of murine erythroleukaemia cells. Biochem. J..

[97] Clarke J.H., Irvine R.F. (2013). Evolutionarily conserved structural changes in phosphatidylinositol 5-phosphate 4-kinase (PI5P4K) isoforms are responsible for differences in enzyme activity and localization. Biochem. J..

[98] Divecha N., Brooksbank C.E.L., Irvine R.F. (1992). Purification and characterization of phosphatidylinositol 4-phosphate 5-kinases. Biochem. J..

[99] Brooksbank C.E.L., Hutchings A., Butcher G.W., Irvine R.F., Divecha N. (1993). Monoclonal antibodies to phosphatidylinositol 4-phosphate 5-kinase: Distribution and intracellular localization of the C isoform. Biochem. J..

[100] Bazenet C.E., Ruano A.R., Brockman J.L., Anderson R.A. (1990). The human erythrocyte contains two forms of phosphatidylinositol-4-phosphate 5-kinase which are differentially active toward membranes. J. Biol. Chem..

[101] Rameh L.E., Tolias K.F., Duckworth B.C., Cantley L.C. (1997). A new pathway for synthesis of phosphatidylinositol-4,5-bisphosphate. Nature.

[102] Divecha N., Truong O., Hsuan J.J., Hinchliffe K.A., Irvine R.F. (1995). The cloning and sequence of the C isoform of PtdIns4*P* 5-kinase. Biochem. J..

[103] Castellino A.M., Parker G.J., Boronenkov I.V., Anderson R.A., Chao M.V. (1997). A Novel Interaction between the Juxtamembrane Region of the p55 Tumor Necrosis Factor Receptor and Phosphatidylinositol-4-phosphate 5-Kinase. J. Biol. Chem..

[104] Boronenkov I.V., Anderson R.A. (1995). The Sequence of Phosphatidylinositol-4-phosphate 5-Kinase Defines a Novel Family of Lipid Kinases. J. Biol. Chem..

[105] Jones D.R., Divecha N. (2004). Linking lipids to chromatin. Curr. Opin. Genet. Dev..

[106] Ciruela A., Hinchliffe K.A., Divecha N., Irvine R.F. (2000). Nuclear targeting of the beta isoform of type II phosphatidylinositol phosphate kinase (phosphatidylinositol 5-phosphate 4-kinase) by its alpha-helix 7. Biochem. J..

[107] Hasegawa J., Strunk B.S., Weisman L.S. (2017). PI5P and PI(3,5)P2: Minor, but Essential Phosphoinositides. Cell Struct. Funct..

[108] Poli A., Zaurito A.E., Abdul-Hamid S., Fiume R., Faenza I., Divecha N. (2019). Phosphatidylinositol 5 Phosphate (PI5P): From Behind the Scenes to the Front (Nuclear) Stage. Int. J. Mol. Sci..

[109] Bulley S.J., Droubi A., Clarke J.H., Anderson K.E., Stephens L.R., Hawkins P.T., Irvine R.F. (2016). In B cells, phosphatidylinositol 5-phosphate 4-kinase–α synthesizes PI(4,5)P2 to impact mTORC2 and Akt signaling. Proc. Natl. Acad. Sci. USA.

[110] Lundquist M.R., Goncalves M.D., Loughran R.M., Possik E., Vijayaraghavan T., Yang A., Pauli C., Ravi A., Verma A., Yang Z. (2018). Phosphatidylinositol-5-Phosphate 4-Kinases Regulate Cellular Lipid Metabolism By Facilitating Autophagy. Mol. Cell.

[111] Vicinanza M., Korolchuk V., Ashkenazi A., Puri C., Menzies F.M., Clarke J., Rubinsztein D.C. (2015). PI(5)P Regulates Autophagosome Biogenesis. Mol. Cell.

[112] Poli A., Pennacchio F.A., Ghisleni A., di Gennaro M., Lecacheur M., Nastały P., Crestani M., Pramotton F.M., Iannelli F., Beznusenko G. (2023). PIP4K2B is mechanoresponsive and controls heterochromatin-driven nuclear softening through UHRF1. Nat. Commun..

[113] Poli A., Abdul-Hamid S., Zaurito A.E., Campagnoli F., Bevilacqua V., Sheth B., Fiume R., Pagani M., Abrignani S., Divecha N. (2021). PIP4Ks impact on PI3K, FOXP3, and UHRF1 signaling and modulate human regulatory T cell proliferation and immunosuppressive activity. Proc. Natl. Acad. Sci. USA.

[114] Blind R.D., Sablin E.P., Kuchenbecker K.M., Chiu H.-J., Deacon A.M., Das D., Fletterick R.J., Ingraham H.A. (2014). The signaling phospholipid PIP _3_ creates a new interaction surface on the nuclear receptor SF-1. Proc. Natl. Acad. Sci. USA.

[115] Ndamukong I., Jones D.R., Lapko H., Divecha N., Avramova Z. (2010). Phosphatidylinositol 5-Phosphate Links Dehydration Stress to the Activity of ARABIDOPSIS TRITHORAX-LIKE Factor ATX1. PLoS ONE.

[116] Sbrissa D., Ikonomov O.C., Shisheva A. (1999). PIKfyve, a Mammalian Ortholog of Yeast Fab1p Lipid Kinase, Synthesizes 5-Phosphoinositides. J. Biol. Chem..

[117] Schaletzky J., Dove S.K., Short B., Lorenzo O., Clague M.J., Barr F.A. (2003). Phosphatidylinositol-5-Phosphate Activation and Conserved Substrate Specificity of the Myotubularin Phosphatidylinositol 3-Phosphatases. Curr. Biol..

[118] Zolov S.N., Bridges D., Zhang Y., Lee W.W., Riehle E., Verma R., Lenk G.M., Converso-Baran K., Weide T., Albin R.L. (2012). In vivo, Pikfyve generates PI(3,5)P_2_, which serves as both a signaling lipid and the major precursor for PI5P. Proc. Natl. Acad. Sci. USA.

[119] Ungewickell A., Hugge C., Kisseleva M., Chang S.C., Zou J., Feng Y., Galyov E.E., Wilson M., Majerus P.W. (2005). The identification and characterization of two phosphatidylinositol-4,5-bisphosphate 4-phosphatases. Proc. Natl. Acad. Sci. USA.

[120] Zou J., Marjanovic J., Kisseleva M.V., Wilson M., Majerus P.W. (2007). Type I phosphatidylinositol-4,5-bisphosphate 4-phosphatase regulates stress-induced apoptosis. Proc. Natl. Acad. Sci. USA.

[121] Posor Y., Eichhorn-Gruenig M., Puchkov D., Schöneberg J., Ullrich A., Lampe A., Müller R., Zarbakhsh S., Gulluni F., Hirsch H. (2013). Spatiotemporal control of endocytosis by phosphatidylinositol-3,4-bisphosphate. Nature.

[122] Alliouachene S., Bilanges B., Chicanne G., Anderson K.E., Pearce W., Ali K., Valet C., Posor Y., Low P.C., Chaussade C. (2015). Inactivation of the Class II PI3K-C2β Potentiates Insulin Signaling and Sensitivity. Cell Rep..

[123] Yokogawa T., Nagata S., Nishio Y., Tsutsumi T., Ihara S., Shirai R., Morita K., Umeda M., Shirai Y., Saitoh N. (2000). Evidence that 3′-phosphorylated polyphosphoinositides are generated at the nuclear surface: Use of immunostaining technique with monoclonal antibodies specific for PI 3,4-P_2_. FEBS Lett..

[124] Marat A.L., Wallroth A., Lo W.T., Müller R., Norata G.D., Falasca M., Schultz C., Haucke V. (2017). mTORC1 activity repression by late endosomal phosphatidylinositol 3,4-bisphosphate. Science.

[125] Gozzelino L., De Santis M.C., Gulluni F., Hirsch E., Martini M. (2020). PI(3,4)P2 Signaling in Cancer and Metabolism. Front. Oncol..

[126] Kerr W.G. (2010). Inhibitor and activator: Dual functions for SHIP in immunity and cancer. Ann. N. Y. Acad. Sci..

[127] Krystal G., Damen J.E., Helgason C.D., Huber M., Hughes M.R., Kalesnikoff J., Lam V., Rosten P., Ware M.D., Yew S. (1999). SHIPs ahoy. Int. J. Biochem. Cell Biol..

[128] Ooms L.M., Binge L.C., Davies E.M., Rahman P., Conway J.R., Gurung R., Ferguson D.T., Papa A., Fedele C.G., Vieusseux J.L. (2015). The Inositol Polyphosphate 5-Phosphatase PIPP Regulates AKT1-Dependent Breast Cancer Growth and Metastasis. Cancer Cell.

[129] Norris F.A., Atkins R.C., Majerus P.W. (1997). The cDNA Cloning and Characterization of Inositol Polyphosphate 4-Phosphatase Type II. J. Biol. Chem..

[130] Watt S.A., Kular G., Fleming I.N., Downes C.P., Lucoqc J.M. (2002). Subcellular localization of phosphatidylinositol 4,5-bisphosphate using the pleckstrin homology domain of phospholipase C δ1. Biochem. J..

[131] Miyazawa A., Umeda M., Horikoshi T., Yanagisawa K., Yoshioka T., Inoue K. (1988). Production and characterization of monoclonal antibodies that bind to phosphatidylinositol 4,5-bisphosphate. Mol. Immunol..

[132] Fukami K., Matsuoka K., Nakanishi O., Yamakawa A., Kawai S., Takenawa T. (1988). Antibody to phosphatidylinositol 4,5-bisphosphate inhibits oncogene-induced mitogenesis. Proc. Natl. Acad. Sci. USA.

[133] Thomas C.L., Steel J., Prestwich G.D., Schiavo G. (1999). Generation of phosphatidylinositol-specific antibodies and their characterization. Biochem. Soc. Trans..

[134] Yildirim S., Castano E., Sobol M., Philimonenko V.V., Dzijak R., Venit T., Hozák P. (2013). Involvement of PIP2 in RNA Polymerase I transcription. J. Cell Sci..

[135] Anderson R.A., Boronenkov I.V., Doughman S.D., Kunz J., Loijens J.C. (1999). Phosphatidylinositol Phosphate Kinases, a Multifaceted Family of Signaling Enzymes. J. Biol. Chem..

[136] Mellman D.L., Gonzales M.L., Song C., Barlow C.A., Wang P., Kendziorski C., Anderson R.A. (2008). A PtdIns4,5P2-regulated nuclear poly(A) polymerase controls expression of select mRNAs. Nature.

[137] Ishihara H., Shibasaki Y., Kizuki N., Wada T., Yazaki Y., Asano T., Oka Y. (1998). Type I phosphatidylinositol-4-phosphate 5-kinases. Cloning of the third isoform and deletion/substitution analysis of members of this novel lipid kinase family. J. Biol. Chem..

[138] Loijens J.C., Boronenkov I.V., Parker G.J., Anderson R.A. (1996). The phosphatidylinositol 4-phosphate 5-kinase family. Adv. Enzym. Regul..

[139] van den Bout I., Divecha N. (2009). PIP5K-driven PtdIns(4,5)P2 synthesis: Regulation and cellular functions. J. Cell Sci..

[140] Tolias K.F., Rameh L.E., Ishihara H., Shibasaki Y., Chen J., Prestwich G.D., Cantley L.C., Carpenter C.L. (1998). Type I Phosphatidylinositol-4-phosphate 5-Kinases Synthesize the Novel Lipids Phosphatidylinositol 3,5-Bisphosphate and Phosphatidylinositol 5-Phosphate. J. Biol. Chem..

[141] Liu Y., Bankaitis V.A. (2010). Phosphoinositide phosphatases in cell biology and disease. Prog. Lipid Res..

[142] Ooms L.M., Horan K.A., Rahman P., Seaton G., Gurung R., Kethesparan D.S., Mitchell C.A. (2009). The role of the inositol polyphosphate 5-phosphatases in cellular function and human disease. Biochem. J..

[143] Dyson J.M., Fedele C.G., Davies E.M., Becanovic J., Mitchell C.A. (2012). Phosphoinositide Phosphatases: Just as Important as the Kinases. Phosphoinosit. I Enzym. Synth. Degrad..

[144] Martelli A.M., Gilmour R.S., Bertagnolo V., Neri L.M. (1992). Nuclear localization and signaling activity of phosphoinositidase C b in Swiss 3T3 cells. Nature.

[145] Song M.S., Salmena L., Pandolfi P.P. (2012). The functions and regulation of the PTEN tumour suppressor. Nat. Rev. Mol. Cell Biol..

[146] Hollander M.C., Blumenthal G.M., Dennis P.A. (2011). PTEN loss in the continuum of common cancers, rare syndromes and mouse models. Nat. Rev. Cancer.

[147] Lindsay Y., McCoull D., Davidson L., Leslie N.R., Fairservice A., Gray A., Lucocq J., Downes C.P. (2006). Localization of agonist-sensitive PtdIns(3,4,5)P3 reveals a nuclear pool that is insensitive to PTEN expression. J. Cell Sci..

[148] Edimo W.E., Vanderwinden J.M., Erneux C. (2013). SHIP2 signalling at the plasma membrane, in the nucleus and at focal contacts. Adv. Biol. Regul..

[149] Foukas L.C., Berenjeno I.M., Gray A., Khwaja A., Vanhaesebroeck B. (2010). Activity of any class IA PI3K isoform can sustain cell proliferation and survival. Proc. Natl. Acad. Sci. USA.

[150] Davis W.J., Lehmann P.Z., Li W. (2015). Nuclear PI3K signaling in cell growth and tumorigenesis. Front. Cell Dev. Biol..

[151] Resnick A.C., Snowman A.M., Kang B.N., Hurt K.J., Snyder S.H., Saiardi A. (2005). Inositol polyphosphate multikinase is a nuclear PI3-kinase with transcriptional regulatory activity. Proc. Natl. Acad. Sci. USA.

[152] Blind R.D., Suzawa M., Ingraham H.A. (2012). Direct Modification and Activation of a Nuclear Receptor–PIP2 Complex by the Inositol Lipid Kinase IPMK. Sci. Signal..

[153] Edimo W.E., Derua R., Janssens V., Nakamura T., Vanderwinden J.M., Waelkens E., Erneux C. (2011). Evidence of SHIP2 Ser132 phosphorylation, its nuclear localization and stability. Biochem. J..

[154] Chung J.-H., Eng C. (2005). Nuclear-Cytoplasmic Partitioning of Phosphatase and Tensin Homologue Deleted on Chromosome 10 (PTEN) Differentially Regulates the Cell Cycle and Apoptosis. Cancer Res..

[155] Liu J.-L., Sheng X., Hortobagyi Z.K., Mao Z., Gallick G.E., Yung W.K.A. (2005). Nuclear PTEN-Mediated Growth Suppression Is Independent of Akt Down-Regulation. Mol. Cell. Biol..

[156] Allis C.D., Jenuwein T. (2016). The molecular hallmarks of epigenetic control. Nat. Rev. Genet..

[157] Lee D.Y., Hayes J.J., Pruss D., Wolffe A.P. (1993). A positive role for histone acetylation in transcription factor access to nucleosomal DNA. Cell.

[158] Biel M., Wascholowski V., Giannis A. (2005). Epigenetics—An Epicenter of Gene Regulation: Histones and Histone-Modifying Enzymes. Angew. Chem. Int. Ed..

[159] McKay L.M., Carpenter B., Roberts S.G. (1999). Regulation of the Wilms’ tumour suppressor protein transcriptional activation domain. Oncogene.

[160] Roberts S.G. (2005). Transcriptional regulation by WT1 in development. Curr. Opin. Genet. Dev..

[161] Carpenter B., Hill K.J., Charalambous M., Wagner K.J., Lahiri D., James D.I., Andersen J.S., Schumacher V., Royer-Pokora B., Mann M. (2004). BASP1 Is a Transcriptional Cosuppressor for the Wilms’ Tumor Suppressor Protein WT1. Mol. Cell. Biol..

[162] Toska E., Campbell H.A., Shandilya J., Goodfellow S.J., Shore P., Medler K.F., Roberts S.G. (2012). Repression of Transcription by WT1-BASP1 Requires the Myristoylation of BASP1 and the PIP2-Dependent Recruitment of Histone Deacetylase. Cell Rep..

[163] Cheng M.K., Shearn A. (2004). The Direct Interaction Between ASH2, a Drosophila Trithorax Group Protein, and SKTL, a Nuclear Phosphatidylinositol 4-Phosphate 5-Kinase, Implies a Role for Phosphatidylinositol 4,5-Bisphosphate in Maintaining Transcriptionally Active Chromatin. Genetics.

[164] Visa N., Percipalle P. (2010). Nuclear Functions of Actin. Cold Spring Harb. Perspect. Biol..

[165] Rando O.J., Zhao K., Janmey P., Crabtree G.R. (2002). Phosphatidylinositol-dependent actin filament binding by the SWI/SNF-like BAF chromatin remodeling complex. Proc. Natl. Acad. Sci. USA.

[166] Batista N.J., Desai S.G., Perez A.M., Finkelstein A., Radigan R., Singh M., Landman A., Drittel B., Abramov D., Ahsan M. (2023). The Molecular and Cellular Basis of Hutchinson–Gilford Progeria Syndrome and Potential Treatments. Genes.

[167] Yu H., Fukami K., Watanabe Y., Ozaki C., Takenawa T. (1998). Phosphatidylinositol 4,5-bisphosphate reverses the inhibition of RNA transcription caused by histone H1. JBIC J. Biol. Inorg. Chem..

[168] Gelato K.A., Tauber M., Ong M.S., Winter S., Hiragami-Hamada K., Sindlinger J., Lemak A., Bultsma Y., Houliston S., Schwarzer D. (2014). Accessibility of Different Histone H3-Binding Domains of UHRF1 Is Allosterically Regulated by Phosphatidylinositol 5-Phosphate. Mol. Cell.

[169] Stijf-Bultsma Y., Sommer L., Tauber M., Baalbaki M., Giardoglou P., Jones D.R., Gelato K.A., van Pelt J., Shah Z., Rahnamoun H. (2015). The Basal Transcription Complex Component TAF3 Transduces Changes in Nuclear Phosphoinositides into Transcriptional Output. Mol. Cell.

[170] Peña P.V., Davrazou F., Shi X., Walter K.L., Verkhusha V.V., Gozani O., Zhao R., Kutateladze T.G. (2006). Molecular mechanism of histone H3K4me3 recognition by plant homeodomain of ING2. Nature.

[171] Li H., Ilin S., Wang W., Duncan E.M., Wysocka J., Allis C.D., Patel D.J. (2006). Molecular basis for site-specific read-out of histone H3K4me3 by the BPTF PHD finger of NURF. Nature.

[172] Feng X., Hara Y., Riabowol K. (2002). Different HATS of the ING1 gene family. Trends Cell Biol..

[173] Jones D.R., Bultsma Y., Keune W.-J., Halstead J.R., Elouarrat D., Mohammed S., Heck A.J., D’Santos C.S., Divecha N. (2006). Nuclear PtdIns5P as a Transducer of Stress Signaling: An In Vivo Role for PIP4Kbeta. Mol. Cell.

[174] Bua D.J., Martin G.M., Binda O., Gozani O. (2013). Nuclear phosphatidylinositol-5-phosphate regulates ING2 stability at discrete chromatin targets in response to DNA damage. Sci. Rep..

[175] Reed S.M., Quelle D.E. (2014). p53 Acetylation: Regulation and Consequences. Cancers.

[176] Nagashima M., Shiseki M., Miura K., Hagiwara K., Linke S.P., Pedeux R., Wang X.W., Yokota J., Riabowol K., Harris C.C. (2001). DNA damage-inducible gene *p33ING2* negatively regulates cell proliferation through acetylation of p53. Proc. Natl. Acad. Sci. USA.

[177] Liu Z., Scannell D.R., Eisen M.B., Tjian R. (2011). Control of Embryonic Stem Cell Lineage Commitment by Core Promoter Factor, TAF3. Cell.

[178] Deato M.D.E., Marr M.T., Sottero T., Inouye C., Hu P., Tjian R. (2008). MyoD Targets TAF3/TRF3 to Activate Myogenin Transcription. Mol. Cell.

[179] Yao J., Fetter R.D., Hu P., Betzig E., Tjian R. (2011). Subnuclear segregation of genes and core promoter factors in myogenesis. Genes Dev..

[180] Mandal P., Eswara K., Yerkesh Z., Kharchenko V., Zandarashvili L., Szczepski K., Bensaddek D., Jaremko Ł., Black B.E., Fischle W. (2022). Molecular basis of hUHRF1 allosteric activation for synergistic histone modification binding by PI5P. Sci. Adv..

[181] Relav L., Doghman-Bouguerra M., Ruggiero C., Muzzi J.C.D., Figueiredo B.C., Lalli E. (2023). Steroidogenic Factor 1, a Goldilocks Transcription Factor from Adrenocortical Organogenesis to Malignancy. Int. J. Mol. Sci..

[182] Krylova I.N., Sablin E.P., Moore J., Xu R.X., Waitt G.M., MacKay J.A., Juzumiene D., Bynum J.M., Madauss K., Montana V. (2005). Structural Analyses Reveal Phosphatidyl Inositols as Ligands for the NR5 Orphan Receptors SF-1 and LRH-1. Cell.

[183] Li Y., Choi M., Cavey G., Daugherty J., Suino K., Kovach A., Bingham N.C., Kliewer S.A., Xu H. (2005). Crystallographic Identification and Functional Characterization of Phospholipids as Ligands for the Orphan Nuclear Receptor Steroidogenic Factor-1. Mol. Cell.

[184] Wang Y.-H., Hariharan A., Bastianello G., Toyama Y., Shivashankar G.V., Foiani M., Sheetz M.P. (2017). DNA damage causes rapid accumulation of phosphoinositides for ATR signaling. Nat. Commun..

[185] Sobol M., Krausová A., Yildirim S., Kalasová I., Fáberová V., Vrkoslav V., Philimonenko V., Marášek P., Pastorek L., Čapek M. (2018). Nuclear phosphatidylinositol 4,5-bisphosphate islets contribute to efficient RNA polymerase II-dependent transcription. J. Cell Sci..

[186] Gavgani F.M., Karlsson T., Tangen I.L., Morovicz A.P., Arnesen V.S., Turcu D.C., Ninzima S., Spang K., Krakstad C., Guillermet-Guibert J. (2021). Nuclear upregulation of class I phosphoinositide 3-kinase p110β correlates with high 47S rRNA levels in cancer cells. J. Cell Sci..

[187] Dyson N.J. (2016). *RB1*: A prototype tumor suppressor and an enigma. Genes Dev..

[188] Kruiswijk F., Labuschagne C.F., Vousden K.H. (2015). p53 in survival, death and metabolic health: A lifeguard with a licence to kill. Nat. Rev. Mol. Cell Biol..

[189] Divecha N., Roefs M., Los A., Halstead J., Bannister A., D’Santos C. (2002). Type I PIPkinases Interact with and Are Regulated by the Retinoblastoma Susceptibility Gene Product—pRB. Curr. Biol..

[190] Los A.P., Vinke F.P., de Widt J., Topham M.K., van Blitterswijk W.J., Divecha N. (2006). The Retinoblastoma Family Proteins Bind to and Activate Diacylglycerol Kinaseζ. J. Biol. Chem..

[191] Los A.P., de Widt J., van Blitterswijk W.J., Divecha N. (2008). Is there a role for diacylglycerol kinase-ζ in cell cycle regulation?. Adv. Enzym. Regul..

[192] Choi S., Chen M., Cryns V.L., Anderson R.A. (2019). A nuclear phosphoinositide kinase complex regulates p53. Nature.

[193] Chen M., Choi S., Wen T., Chen C., Thapa N., Lee J.H., Cryns V.L., Anderson R.A. (2022). A p53–phosphoinositide signalosome regulates nuclear AKT activation. Nature.

[194] Ahmed N.N., Franke T.F., Bellacosa A., Datta K., Gonzalez-Portal M.E., Taguchi T., Testa J.R., Tsichlis P.N. (1993). The proteins encoded by c-akt and v-akt differ in post-translational modification, subcellular localization and oncogenic potential. Oncogene.

[195] Neri L.M., Milani D., Bertolaso L., Stroscio M., Bertagnolo V., Capitani S. (1994). Nuclear translocation of phosphatidylinositol 3-kinase in rat pheochromocytoma PC 12 cells after treatment with nerve growth factor. Cell. Mol. Biol..

[196] Nguyen T.L.X., Choi J.W., Lee S.B., Ye K., Woo S.-D., Lee K.-H., Ahn J.-Y. (2006). Akt phosphorylation is essential for nuclear translocation and retention in NGF-stimulated PC12 cells. Biochem. Biophys. Res. Commun..

[197] Stracker T.H., Petrini J.H.J. (2011). The MRE11 complex: Starting from the ends. Nat. Rev. Mol. Cell Biol..

[198] Branzei D., Foiani M. (2008). Regulation of DNA repair throughout the cell cycle. Nat. Rev. Mol. Cell Biol..

[199] Jones D.R., Foulger R., Keune W., Bultsma Y., Divecha N. (2012). PtdIns5 *P* is an oxidative stress-induced second messenger that regulates PKB activation. FASEB J..

[200] Keune W.-J., Jones D.R., Bultsma Y., Sommer L., Zhou X.Z., Lu K.P., Divecha N. (2012). Regulation of Phosphatidylinositol-5-Phosphate Signaling by Pin1 Determines Sensitivity to Oxidative Stress. Sci. Signal..

[201] Bunce M.W., Boronenkov I.V., Anderson R.A. (2008). Coordinated Activation of the Nuclear Ubiquitin Ligase Cul3-SPOP by the Generation of Phosphatidylinositol 5-Phosphate. J. Biol. Chem..

[202] Xu L., Wei Y., Reboul J., Vaglio P., Shin T.-H., Vidal M., Elledge S.J., Harper J.W. (2003). BTB proteins are substrate-specific adaptors in an SCF-like modular ubiquitin ligase containing CUL-3. Nature.

[203] Geyer R., Wee S., Anderson S., Yates J., Wolf D.A. (2003). BTB/POZ Domain Proteins Are Putative Substrate Adaptors for Cullin 3 Ubiquitin Ligases. Mol. Cell.

[204] Li X.-M., Wu H.-L., Xia Q.-D., Zhou P., Wang S.-G., Yu X., Hu J. (2022). Novel insights into the SPOP E3 ubiquitin ligase: From the regulation of molecular mechanisms to tumorigenesis. Biomed. Pharmacother..

[205] Okada M., Jang S.-W., Ye K. (2008). Akt phosphorylation and nuclear phosphoinositide association mediate mRNA export and cell proliferation activities by ALY. Proc. Natl. Acad. Sci. USA.

[206] Mohan N., AP S., Francis N., Anderson R., Laishram R.S. (2015). Phosphorylation regulates the Star-PAP-PIPKIα interaction and directs specificity toward mRNA targets. Nucleic Acids Res..

[207] Gonzales M.L., Mellman D.L., Anderson R.A. (2008). CKIα Is Associated with and Phosphorylates Star-PAP and Is Also Required for Expression of Select Star-PAP Target Messenger RNAs. J. Biol. Chem..

[208] Zhang L., Zhang Y., Chen Y., Gholamalamdari O., Wang Y., Ma J., Belmont A.S. (2020). TSA-seq reveals a largely conserved genome organization relative to nuclear speckles with small position changes tightly correlated with gene expression changes. Genome Res..

[209] Xu A., Suh P.-G., Marmy-Conus N., Pearson R.B., Seok O.Y., Cocco L., Gilmour R.S. (2001). Phosphorylation of Nuclear Phospholipase C β1 by Extracellular Signal-Regulated Kinase Mediates the Mitogenic Action of Insulin-Like Growth Factor I. Mol. Cell. Biol..

[210] Xu A., Wang Y., Xu L.Y., Gilmour R.S. (2001). Protein Kinase C α-mediated Negative Feedback Regulation Is Responsible for the Termination of Insulin-like Growth Factor I-induced Activation of Nuclear Phospholipase C β1 in Swiss 3T3 Cells. J. Biol. Chem..

